# Coordinate Nuclear Targeting of the FANCD2 and FANCI Proteins *via* a FANCD2 Nuclear Localization Signal

**DOI:** 10.1371/journal.pone.0081387

**Published:** 2013-11-21

**Authors:** Rebecca A. Boisvert, Meghan A. Rego, Paul A. Azzinaro, Maurizio Mauro, Niall G. Howlett

**Affiliations:** 1 Department of Cell and Molecular Biology, University of Rhode Island, Kingston, Rhode Island, United States of America; 2 Department of Genetics, Harvard Medical School, Boston, Massachusetts, United States of America; 3 Department of Obstetrics & Gynecology and Women's Health, Albert Einstein College of Medicine, Bronx, New York, United States of America; University of Minnesota, United States of America

## Abstract

Fanconi anemia (FA) is a rare recessive disease, characterized by congenital defects, bone marrow failure, and increased cancer susceptibility. FA is caused by biallelic mutation of any one of sixteen genes. The protein products of these genes function cooperatively in the FA-BRCA pathway to repair DNA interstrand crosslinks (ICLs). A central step in the activation of this pathway is the monoubiquitination of the FANCD2 and FANCI proteins. Monoubiquitinated FANCD2 and FANCI localize to discrete chromatin regions where they function in ICL repair. Despite their critical role in ICL repair, very little is known about the structure, function, and regulation of the FANCD2 and FANCI proteins, or how they are targeted to the nucleus and chromatin. In this study, we describe the functional characterization of an amino-terminal FANCD2 nuclear localization signal (NLS). We demonstrate that the amino terminal 58 amino acids of FANCD2 can promote the nuclear expression of GFP and is necessary for the nuclear localization of FANCD2. Importantly, mutation of this FANCD2 NLS reveals that intact FANCD2 is required for the nuclear localization of a subset of FANCI. In addition, the NLS is necessary for the efficient monoubiquitination of FANCD2 and FANCI and, consequently, for their localization to chromatin. As a result, FANCD2 NLS mutants fail to rescue the ICL sensitivity of FA-D2 patient cells. Our studies yield important insight into the domain structure of the poorly characterized FANCD2 protein, and reveal a previously unknown mechanism for the coordinate nuclear import of a subset of FANCD2 and FANCI, a key early step in the cellular ICL response.

## Introduction

Fanconi anemia (FA) is a rare autosomal and X-linked recessive disease, characterized by congenital abnormalities, pediatric bone marrow failure, and heightened cancer susceptibility [1]. FA is caused by biallelic mutations in any one of 16 genes (*FANCA, -B, -C, -D1/BRCA2, -D2, -E, -F, -G, -I, -J/BRIP1, -L, -M, -N/PALB2, -P/SLX4, -O/RAD51C, -Q/ERCC4*) [2-4]. The protein products of these genes function cooperatively in the FA-BRCA pathway to repair DNA interstrand crosslinks (ICLs) and to maintain chromosome stability. A central step in the activation of the FA-BRCA pathway is the monoubiquitination of the FANCD2 and FANCI proteins. Monoubiquitination of FANCD2 and FANCI is catalyzed by the FA core complex, which is comprised of FANCA, FANCB, FANCC, FANCE, FANCF, FANCG, FANCL, and FANCM, in addition to the E2 ubiquitin conjugating enzyme UBE2T, as well as FAAP100, FAAP24, and FAAP20 [4,5]. Following monoubiquitination, FANCD2 and FANCI co-localize in discrete nuclear foci with several well characterized DNA repair proteins including BRCA1 and NBS1 [6-9]. However, importantly, the domain structure, regulation, and function of both FANCD2 and FANCI are poorly described. Furthermore, the mechanism(s) by which FANCD2 and FANCI are localized to the nucleus remains unknown.

Several studies have established that the FANCD2 and FANCI proteins function proximal to or within chromatin [6,8-10]. As FANCD2 and FANCI are relatively large proteins, with molecular weights of 164 and 149 kDa, respectively, the nuclear import of these proteins necessitates an energy-dependent active transport mechanism. The most common nuclear protein transport mechanism involves the recognition of nuclear localization signals (NLSs) by members of the importin (Imp) superfamily of nuclear transporters, followed by translocation through the nuclear pore complexes [11]. NLSs generally comprise short stretches of basic amino acids either alone (monopartite) or separated by one (bipartite) or two (tripartite) mutation-tolerant linker regions of 10-12 amino acids. NLSs in the cargo protein are recognized by the Imp α subunit of the Imp α/β heterodimer or by Imp β alone, and nuclear transport is coupled to GTP hydrolysis [11]. 

In this study, we describe the identification and functional characterization of a NLS in the amino terminus of FANCD2. We demonstrate that the amino terminal 58 amino acids of FANCD2 can promote the nuclear expression of green fluorescent protein (GFP). In addition, using deletion and site-directed mutagenesis strategies we establish that the amino terminal 58 amino acids of FANCD2 are necessary for the nuclear and chromatin localization of FANCD2 in FA-D2 patient-derived cells. Importantly, we also demonstrate that the nuclear and chromatin localization of a subset of the cellular pool of FANCI is dependent on the nuclear import of FANCD2. Moreover, the FANCD2 NLS is required for the efficient DNA damage-inducible monoubiquitination of FANCD2 and FANCI. Consequently, the FANCD2 NLS mutants fail to rescue the ICL hypersensitivity of FA-D2 patient-derived cells. Our results suggest that a subset of FANCI is translocated to the nucleus in a piggyback mechanism with FANCD2, dependent on its amino-terminal NLS, and suggest that FANCD2 and FANCI are targeted to the nucleus as a heterodimer. These findings lend important insight into the structure and regulation of two poorly characterized tumor suppressor proteins with key early roles in the cellular ICL response.

## Results

### FANCD2 contains a highly conserved amino-terminal nuclear localization signal, which facilitates nuclear expression of GFP

**Figure 1:**
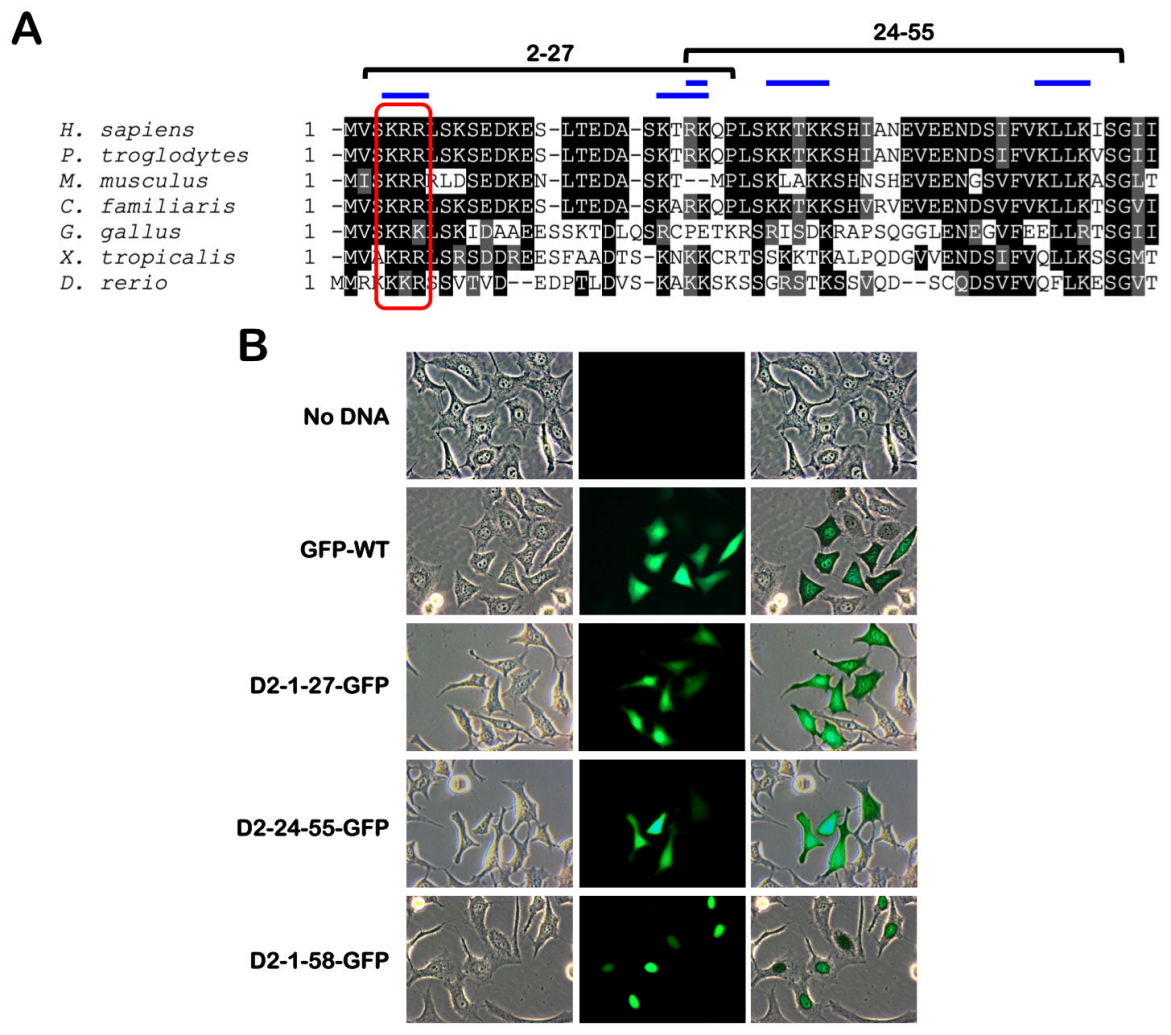
The amino terminal 58 amino acids of FANCD2 contain a highly conserved nuclear localization signal, which facilitates nuclear expression of GFP. (**A**) A Clustal Omega multiple sequence alignment of the amino terminus of FANCD2 demonstrates strong evolutionary conservation of several blocks of basic amino acids of this region, indicated by blue bars. The red box indicates the highly conserved K4, R5, and R6 residues selected for mutagenesis. (**B**) The amino terminal 58 amino acids of FANCD2 are required to promote exclusive nuclear expression of GFP. HeLa cells were transiently transfected with wild type GFP (GFP-WT), amino acids 1-27 (D2-1-27-GFP), 24-55 (D2-24-55-GFP), or 1-58 (D2-1-58-GFP) of FANCD2 fused to GFP followed by analysis by inverted fluorescence microscopy.

*In silico* analysis using cNLS mapper uncovered several high-scoring Imp α/β-dependent bipartite NLSs within the amino-terminal 58 amino acids of FANCD2 (Figs. **S1A and B**) [12]. In contrast, cNLS mapper did not predict any high scoring NLSs in FANCI. A sequence alignment of FANCD2 from multiple species illustrates strong evolutionary conservation in general (Fig. **S1C**). However, in contrast to the sequence divergent carboxy-terminus, the alignment illustrates strong conservation of several blocks of basic amino acids within the amino terminal 58 amino acids (Figs. **1A** and **S1C**). To determine if this entire region or amino acids 1-27 or 24-55 - the two highest scoring predicted NLSs (Fig. **S1B**) - were sufficient for nuclear localization, we fused amino acids 1-58 (D2-1-58-GFP), 1-27 (D2-1-27-GFP), or 24-55 (D2-24-55-GFP) of FANCD2 to the amino terminus of GFP (Fig. **1B**). HeLa cells were transiently transfected with wild type GFP (GFP-WT) and the FANCD2-GFP fusion constructs and cells were analyzed by inverted fluorescence microscopy. HeLa cells transiently expressing GFP-WT, D2-1-27-GFP, or D2-24-55-GFP all exhibited uniform cytoplasmic and nuclear fluorescence (Fig. **1B**). Conversely, cells transiently expressing D2-1-58-GFP primarily exhibited nuclear fluorescence (Fig. **1B**). Similar findings were observed with IMR90 cells (Fig. **S1D**). These results demonstrate that the amino terminal 58 amino acids of FANCD2 are necessary to promote exclusive nuclear GFP localization. In support of an importin α/β-dependent mechanism of nuclear import, treatment with ivermectin, a broad-spectrum inhibitor of importin α/β-dependent nuclear import [13], inhibited the exclusive nuclear localization of D2-1-58-GFP, herein referred to as D2-NLS-GFP (Figs. **S1E and F**). In addition, mass spectrometry analysis of FANCD2 immune complexes revealed the presence of importin β1, as well as the nuclear pore complex proteins NUP160 and NUP155 (Table **S1**). Using a chromatin fractionation approach we also observed that the majority of GFP-WT resided in a soluble cytoplasmic and nuclear fraction (S) (Figure **S1G** **, lane 5**). While a large proportion of D2-NLS-GFP also resided in a soluble cytoplasmic and nuclear fraction, a higher relative proportion of D2-NLS-GFP was detected in a chromatin-associated nuclear fraction (C) (Figures **S1G** **, lane 9 and H**). Taken together these results demonstrate that the amino-terminal 58 amino acids of FANCD2 harbor a *bona fide* NLS that can promote exclusive nuclear GFP localization. 

### The FANCD2 NLS is required for the nuclear localization of FANCD2

**Figure 2:**
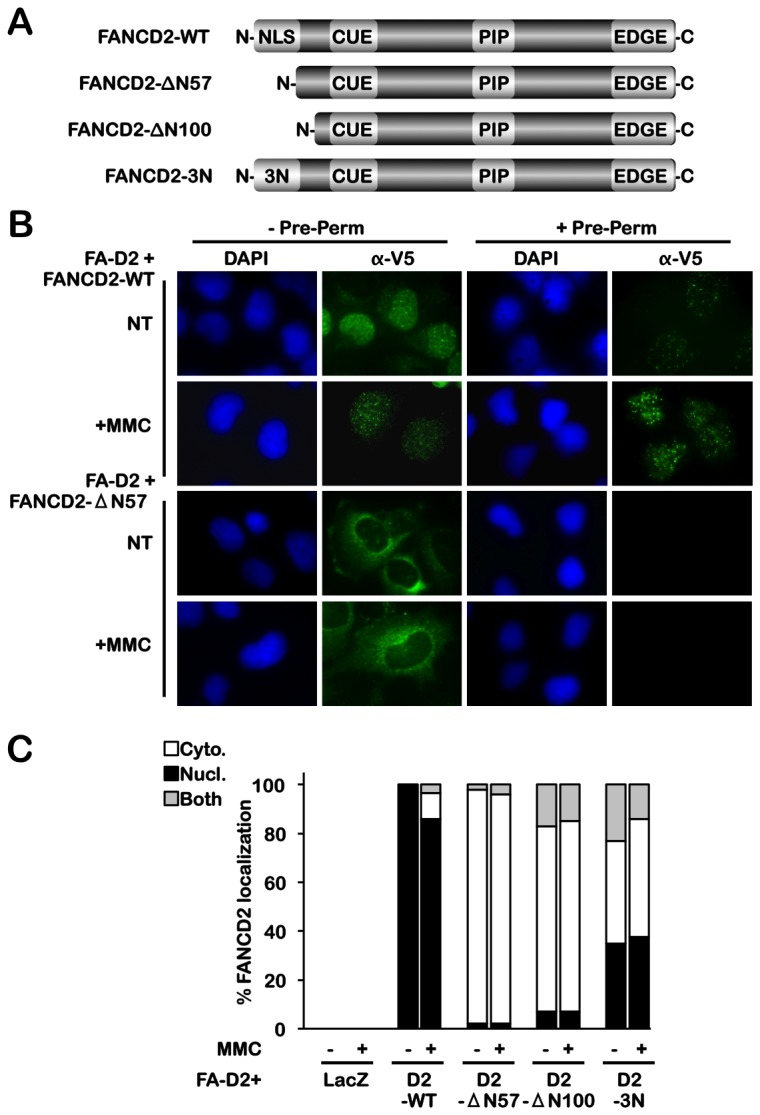
The FANCD2 NLS is required for the nuclear localization of FANCD2. (**A**) Schematic diagram of the FANCD2 constructs generated in this study. In addition to the NLS, FANCD2 harbors a CUE (for coupling of ubiquitin conjugation to endoplasmic reticulum degradation) ubiquitin-binding domain [47], a PCNA-interaction motif or PIP-box [19], and a carboxy-terminus EDGE motif [10]. (**B**) FA-D2 cells stably expressing FANCD2-WT and FANCD2-∆N57 were incubated in the absence (NT) or presence of 40 nM MMC for 24 h. Cells were then incubated in the absence (-Pre-Perm) or presence (+Pre-Perm) of pre-permeablization buffer, fixed, stained with mouse monoclonal anti-V5 antibody (green) and counterstained with DAPI (blue). (**C**) FA-D2 cells stably expressing LacZ, FANCD2-WT, FANCD2-∆N57, FANCD2-∆N100, and FANCD2-3N were incubated in the absence (NT) or presence of MMC for 24 h, fixed, and stained with rabbit polyclonal anti-FANCD2 antibody, and counterstained with phalloidin and DAPI. At least 300 cells were scored for cytoplasmic (Cyto.), nuclear (Nucl.), and both cytoplasmic and nuclear (Both) localization of FANCD2.

To determine the functional significance of the FANCD2 NLS, we next generated deletion and missense mutations of this amino acid sequence. Two amino-terminal deletion mutations, FANCD2-ΔN57, lacking amino acids 2-58, and FANCD2-ΔN100, lacking amino acids 2-101, were generated (Figure **2A**). In addition, using a site-directed mutagenesis approach, amino acids K4, R5, and R6, the most highly conserved basic amino acids within this region (Figure **1A**), were mutated to N4, N5, and N6, herein referred to as FANCD2-3N (Figure **2A**). These *FANCD2* cDNAs were cloned into the pLenti6.2 lentiviral vector, which contains a carboxy-terminal V5 tag, and lentivirus was used to generate a series of PD20 FA-D2 patient-derived cells stably expressing wild type or mutant FANCD2. These FA-D2 cells harbor a maternally inherited A-G change at nucleotide 376 that leads to the production of a severely truncated protein, and a paternally inherited missense hypomorphic mutation leading to a R1236H change [14]. Immunofluorescence microscopy (IF) revealed that deletion of the FANCD2 NLS resulted in exclusive cytoplasmic localization of FANCD2, in contrast to wild type FANCD2, which exhibited both diffuse and focal nuclear localization (Figures **2B and C**). Furthermore, permeabilization of FA-D2 cells expressing the FANCD2-ΔN57 mutant with non-ionic detergent resulted in complete loss of fluorescent signal indicating the high solubility of cytoplasmic FANCD2-ΔN57 (Figure **2B**). In contrast, nuclear and focal localization of wild type FANCD2 was largely resistant to permeabilization (Figure **2B**). Similar findings were obtained with FA-D2 cells expressing FANCD2-ΔN100 (Figure **2C**). In addition, a partial yet significant defect in nuclear localization was observed for the FANCD2-3N mutant, compared to wild type FANCD2 (Figure **2C**). 

### The FANCD2 NLS is required for the nuclear localization of a subset of FANCI

**Figure 3:**
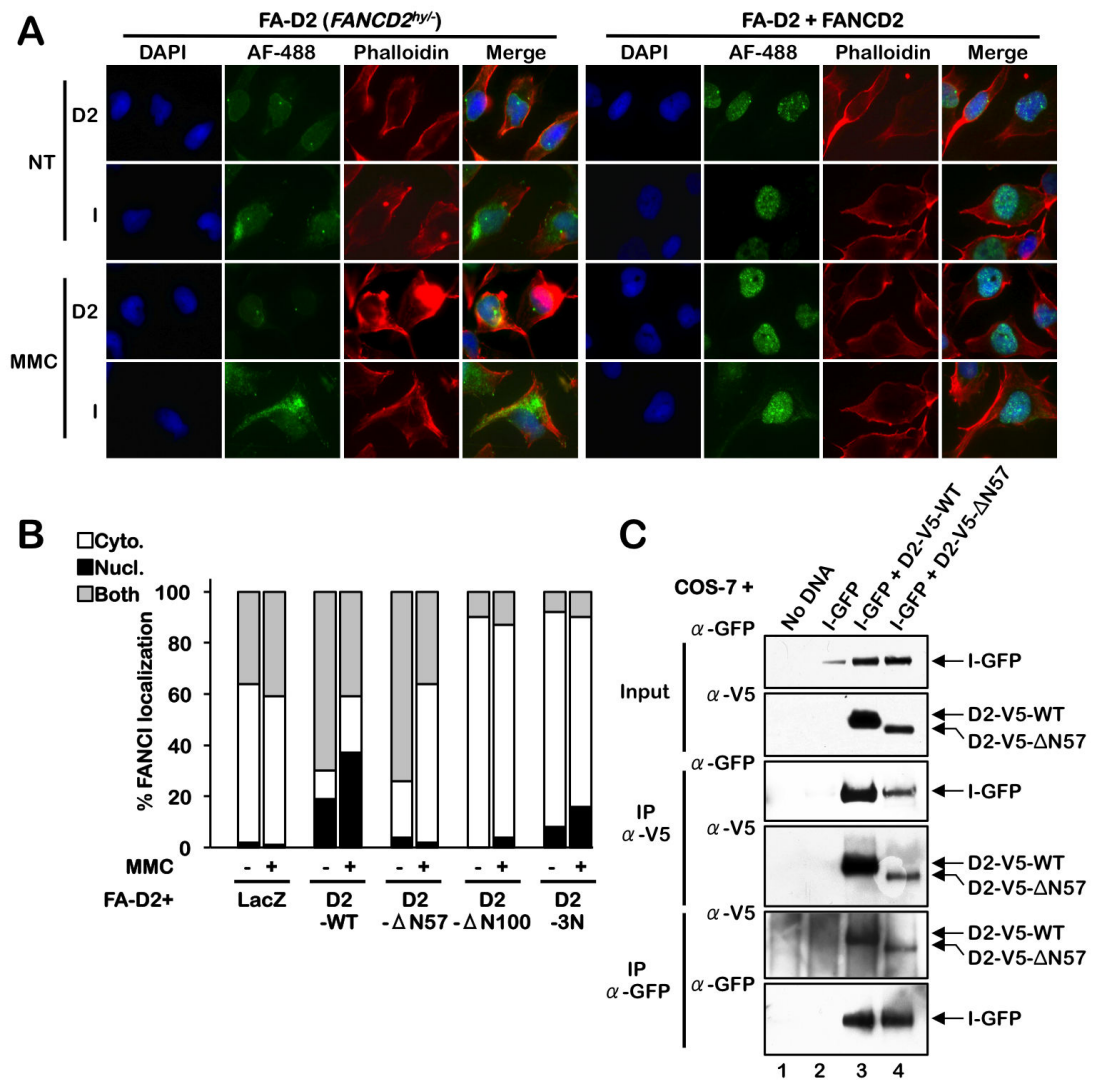
The FANCD2 NLS is required for the nuclear localization of a subset of FANCI. (**A**) FA-D2 patient cells or FA-D2 cells stably expressing FANCD2-WT were incubated in the absence (NT) or presence of MMC for 24 h, fixed, stained with rabbit polyclonal anti-FANCD2 or anti-FANCI antibody and counterstained with phalloidin and DAPI. AF-488, Alexa Fluor 488. (**B**) FA-D2 cells stably expressing LacZ, FANCD2-WT, FANCD2-∆N57, FANCD2-∆N100, and FANCD2-3N were incubated in the absence (NT) or presence of MMC for 24 h, fixed, and stained with rabbit polyclonal anti-FANCI antibody, and counterstained with phalloidin and DAPI. At least 300 cells were scored for cytoplasmic (Cyto.), nuclear (Nucl.), and both cytoplasmic and nuclear (Both) localization of FANCI. (**C**) COS-7 cells were transiently transfected with no DNA, FANCI-GFP, FANCI-GFP plus FANCD2-V5-WT, or FANCI-GFP plus FANCD2-V5-∆N57. Whole-cell lysates were immunoprecipitated with anti-V5 or anti-GFP antibodies and immune complexes immunoblotted with anti-GFP and anti-V5 antibodies.

Next, we examined the sub-cellular localization of FANCI in our FA-D2 cell series. It is important to note that the FA-D2 cells used in this study, like all known FA-D2 patient-derived lines, harbor hypomorphic *FANCD2* mutations and express residual FANCD2 protein [15]. IF analysis revealed that FANCI was uniformly present in both the cytoplasm and nucleus of FA-D2 cells in the absence or presence of MMC (Figures **3A and B**). Importantly, complementation of FA-D2 cells with wild type FANCD2 (FANCD2-WT) led to a large increase in exclusive nuclear localization of FANCI (Figures **3A and B**). Furthermore, MMC-inducible FANCI nuclear foci formation was restored in these cells, consistent with previous studies [8,9] (Figure **3A**). Similar results were obtained with hTERT-immortalized mutant and FANCD2-complemented KEAE FA-D2 patient-derived cells [15] (Figure **S2A**). In contrast to wild type FANCD2, both the FANCD2-ΔN57 and -ΔN100 NLS mutants failed to promote the exclusive nuclear localization of FANCI (Figures **3B** and **S2B**). Furthermore, a partial defect in FANCI nuclear localization was observed for FA-D2 cells expressing FANCD2-3N (Figure **3B**). To rule out the possibility that deletion of the amino-terminus of FANCD2 prevented its heterodimerization with FANCI, we tested the ability of the FANCD2-ΔN57 mutant to physically interact with FANCI by transiently transfecting COS-7 cells with GFP-tagged FANCI and V5-tagged FANCD2-WT or FANCD2-ΔN57 and examining their ability to interact by co-immunoprecipitation. Co-expression of both FANCD2-WT and FANCD2-ΔN57 with FANCI led to its stabilization (Figure **3C** **, upper panel, lanes 3 and 4**). Furthermore, the FANCD2-ΔN57 mutant was capable of interacting with FANCI and, when corrected for levels of protein input, no appreciable difference in the level of interaction between FANCI and FANCD2-ΔN57 and FANCI and FANCD2-WT was observed (Figure **3C** **, lower panels, lanes 3 and 4**). Together, these results establish that the nuclear localization of a subset of FANCI is dependent in part on FANCD2, and suggest a piggyback mechanism of nuclear FANCI targeting that is dependent on the FANCD2 amino-terminal NLS. 

### The FANCD2 NLS is required for efficient FANCD2 and FANCI monoubiquitination and chromatin association

**Figure 4:**
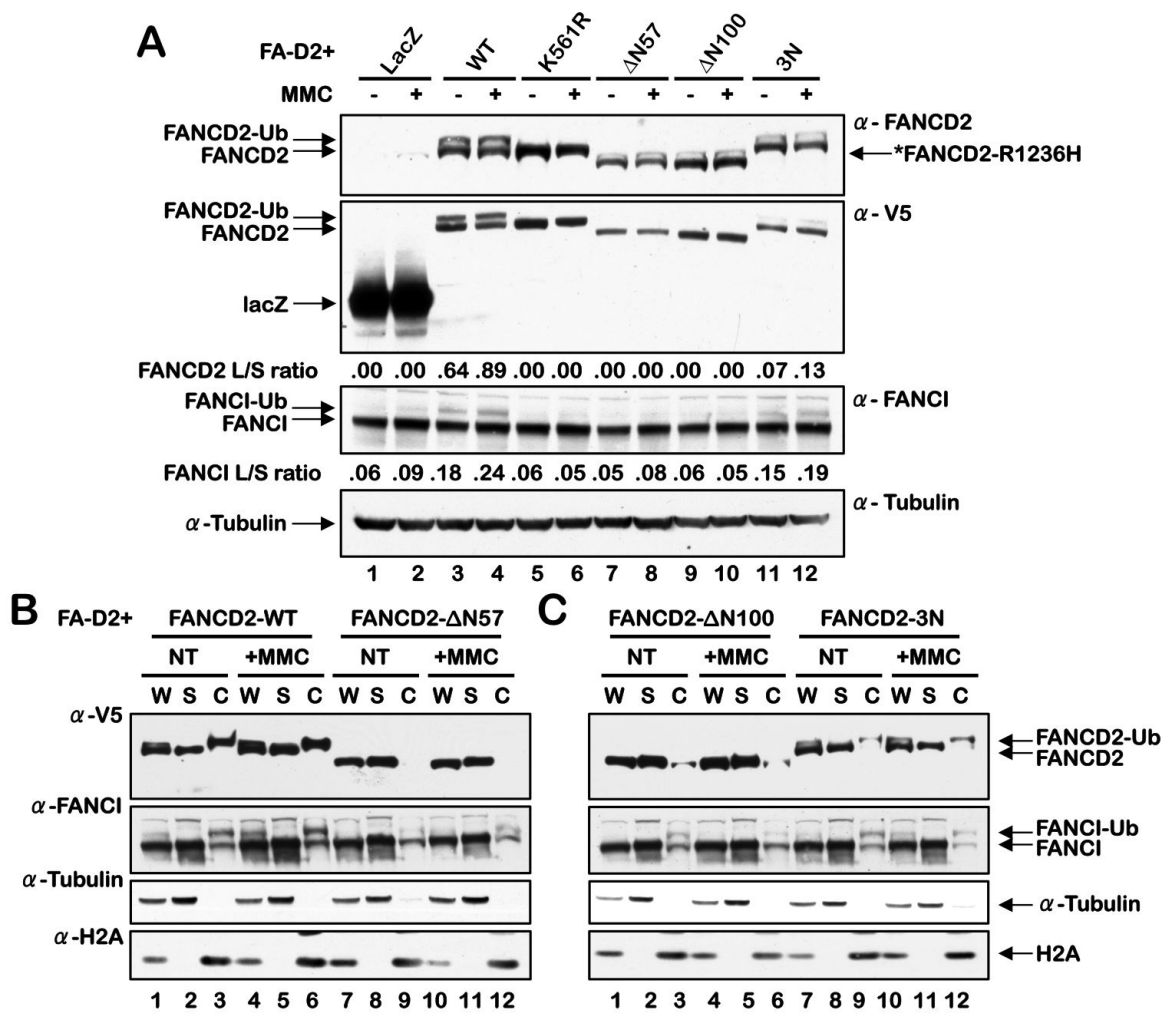
The FANCD2 NLS is required for efficient FANCD2 and FANCI monoubiquitination and chromatin association. (**A**) FA-D2 cells stably expressing LacZ, FANCD2-WT, FANCD2-K561R, FANCD2-∆N57, FANCD2-∆N100 and FANCD2-3N were incubated in the absence and presence of 250 nM MMC for 18 h, and whole-cell lysates were immunoblotted with antibodies to FANCD2, V5, FANCI and α-tubulin. The FANCD2 and FANCI L/S ratios are the ratios of monoubiquitinated to nonubiquitinated protein, and were calculated by measuring protein band intensities using ImageJ image processing and analysis software (http://rsb.info.nih.gov/ij/). (**B** and **C**) FA-D2 cells stably expressing FANCD2-WT, FANCD2-∆N57, FANCD2-∆N100 and FANCD2-3N were treated as above and cell pellets were fractionated into soluble (S) and chromatin-associated (C) fractions. Fractions were immunoblotted with antibodies against V5, FANCI, α-tubulin and H2A. W, unfractionated whole cell extract.

Next, we examined the consequences of disruption of the FANCD2 NLS on the monoubiquitination and chromatin localization of FANCD2 and FANCI. Similar to the FANCD2-K561R mutant that cannot be monoubiquitinated [6], mutation of the FANCD2 NLS had a significant impact on its monoubiquitination: the ΔN57 and ΔN100 NLS deletion mutants failed to undergo spontaneous or MMC-inducible FANCD2 monoubiquitination (Figure **4A** **, lanes 7-10**). A faint band of similar molecular weight to wild type FANCD2 can be seen in the α-FANCD2 panel for FA-D2 cells expressing FANCD2-ΔN57 and -ΔN100 (Figure **4A** **, lanes 7-10**). This is most likely the hypomorphic FANCD2-R1236H missense mutant (*see Materials and Methods*) [14], as this band is also present in FA-D2 cells expressing LacZ (lanes 1 and 2) and is not recognized by the anti-V5 antibody (Figure **4** **, lanes 7-10**). The FANCD2-3N mutant also exhibited considerably reduced spontaneous and MMC-inducible monoubiquitination (Figure **4A** **, lanes 11 and 12**). Previous studies have demonstrated that FANCD2 and FANCI monoubiquitination are interdependent [8,9]. Accordingly, FANCI monoubiquitination was largely abrogated in FA-D2 cells stably expressing the ΔN57 and ΔN100 NLS deletion mutants, similar to that observed in cells expressing FANCD2-K561R (Figure **4A** **, lanes 5-10**). A very modest statistically insignificant decrease in FANCI monoubiquitination was also observed for FA-D2 cells expressing the FANCD2-3N mutant (Figure **4A** **, lanes 11 and 12**). In addition, a chromatin fractionation approach revealed that the FANCD2-ΔN57, -ΔN100, and -3N mutants were largely defective in chromatin localization compared to FANCD2-WT (Figures **4B and C**). Furthermore, while robust FANCI chromatin localization was observed for cells expressing FANCD2-WT, in comparison, FANCI chromatin localization was considerably diminished in cells expressing the FANCD2-ΔN57, -ΔN100, and -3N mutants (Figures **4B and C and S3**). It is important to note that this particular fractionation protocol does not distinguish between true chromatin and the nuclear matrix. 

### The FANCD2 NLS mutants fail to correct the ICL sensitivity of FA-D2 patient cells

**Figure 5:**
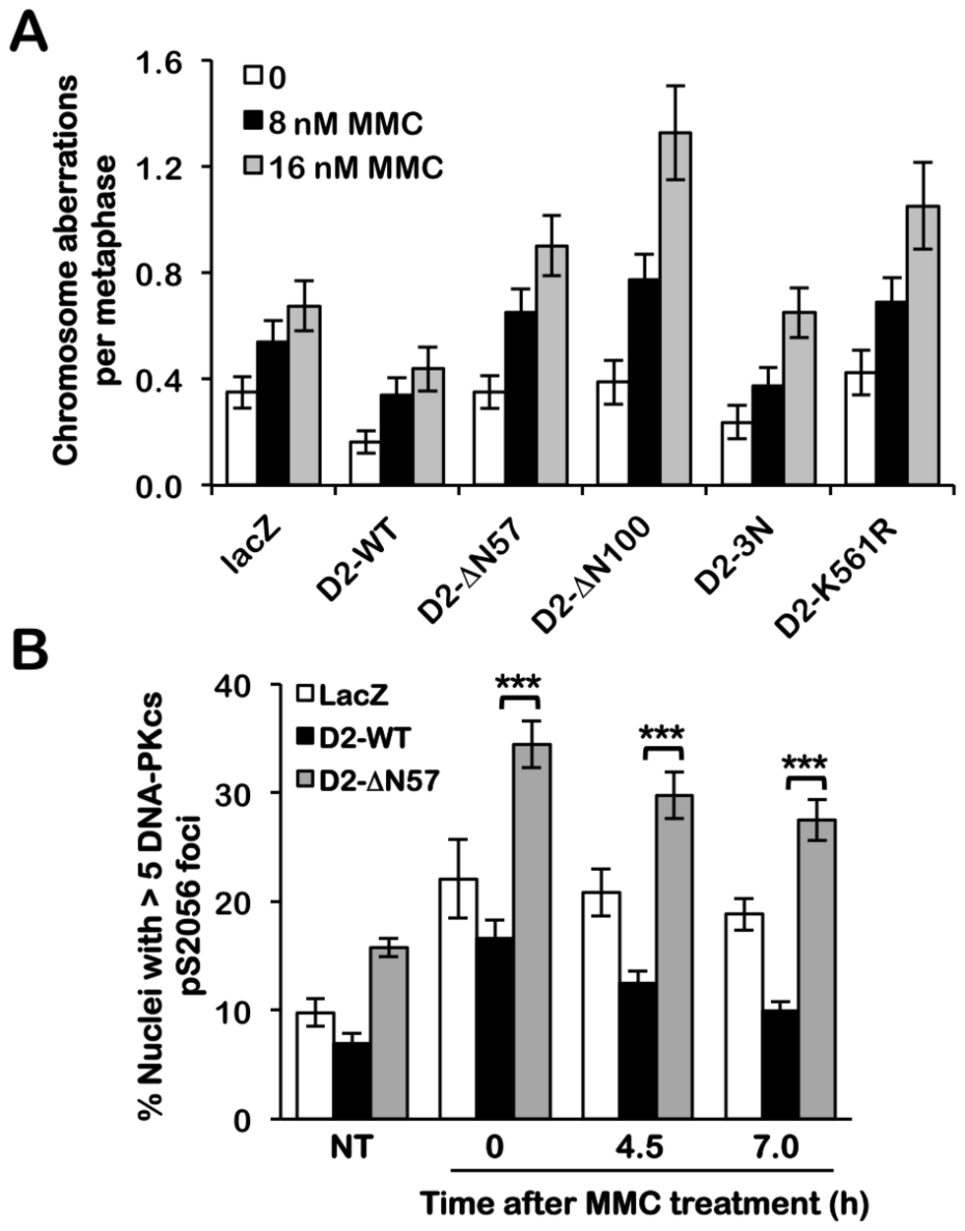
The FANCD2 NLS mutants fail to correct the ICL sensitivity of FA-D2 patient cells. (**A**) FA-D2 cells stably expressing LacZ, FANCD2-WT, FANCD2-∆N57, FANCD2-∆N100, FANCD2-3N, or FANCD2-K561R were incubated in the absence or presence of 8 or 16 nM MMC for 24 h and the numbers of chromosome aberrations including gaps and breaks, dicentrics, and complex chromosome aberrations, including radial formations, were scored. Metaphase spreads were analyzed using a Zeiss AxioImager.A1 upright epifluorescent microscope with AxioVision LE 4.6 image acquisition software. At least 80 metaphases were scored per treatment. Error bars represent the standard error of the means. (**B**) FA-D2 cells stably expressing LacZ, FANCD2-WT and FANCD2-∆N57 were incubated in the absence (NT) or presence of 40 nM MMC for 18 h, and allowed to recover for 0, 4.5 or 7 h. Cells were then fixed, stained with rabbit polyclonal anti-DNA-PK_CS_ pS2056, and counterstained with DAPI. At least 300 cells were scored for nuclei with > 5 DNA-PK_CS_ foci. Error bars represent the standard error of the means from two independent experiments. ***, *p* < 0.001.

FA patient-derived cells are hypersensitive to ICL-induced cytotoxicity and clastogenicity [16]. Therefore, we next assessed the ability of the FANCD2 NLS mutants to rescue the MMC sensitivity of FA-D2 cells. In a MMC clastogenicity assay, FA-D2 cells expressing the ΔN57 and ΔN100 NLS mutants exhibited markedly elevated levels of chromosome aberrations, including gaps and breaks, dicentrics, and radial formations, compared with FA-D2 cells complemented with wild type FANCD2 (*p* = 0.001 and 1.1 x 10^-5^, respectively for 16 nM MMC) (Figure **5A**). In addition, in a MMC cytotoxicity assay, similar to FA-D2 cells expressing FANCD2-K561R [6], cells expressing the FANCD2-ΔN57 and -ΔN100 NLS mutants displayed increased MMC sensitivity compared with cells expressing wild type FANCD2 (*p* < 0.05 at 50 nM for FANCD2-ΔN57, -ΔN100, and -K561R compared with wild type FANCD2) (Figure **S4**). Recent studies have demonstrated increased error-prone nonhomologous DNA end joining (NHEJ) in FA patient cells [17]. Therefore, we also examined the recruitment of DNA-PK_CS_ to nuclear foci in FA-D2 cells expressing LacZ, wild type FANCD2 or the FANCD2 ΔN57 mutant, using an antibody raised against DNA-PK_CS_ phosphorylated on S2056, a marker of NHEJ [17,18]. Persistent increased DNA-PK_CS_ pS2056 nuclear foci formation was observed in FA-D2 cells expressing LacZ following treatment with MMC, and this phenotype was rescued by wild type FANCD2 (Figure **5B**). In contrast, we observed markedly increased MMC-inducible DNA-PK_CS_ pS2056 nuclear foci formation in FA-D2 cells expressing FANCD2 ΔN57, compared to cells expressing LacZ or wild type FANCD2 (Figure **5B**). Taken together, these results demonstrate that the FANCD2 NLS is essential for the correct function of the FA-BRCA pathway in the cellular ICL response.

## Discussion

Despite their critical role in the cellular ICL response and their tumor suppressor function, very little is known about the structure, function, and regulation of the FANCD2 and FANCI proteins. For FANCD2, a large 1451 amino acid protein, only two functional motifs, a PCNA-interaction motif, or PIP box, and a carboxy-terminus EDGE motif, have been described to date [10,19]. Our laboratory has also recently identified and characterized a CUE ubiquitin-binding domain in the amino-terminus of FANCD2, which mediates noncovalent binding to ubiquitin, and is essential for efficient cellular ICL repair [20]. In this study we describe the functional characterization of an amino terminal FANCD2 NLS. While a previous study reported the existence of a FANCD2 NLS, this study failed to examine the functional consequences of its disruption in a FA-D2 patient-derived cell system [21]. Here, we demonstrate that fusion of amino acids 1-58 of FANCD2 to the amino terminus of GFP drives its exclusive nuclear localization. cNLS mapper identified several high scoring putative bipartite NLSs within this region, in particular, amino acids 2-27 and 24-55. However, in contrast to amino acids 1-58, fusion of either sequence to GFP failed to drive exclusive nuclear GFP expression. Furthermore, mutation of K4, R5, and R6, the most highly conserved block of basic amino acids within this region, reduced, but did not abrogate, nuclear FANCD2 localization in FA-D2 cells. These results establish that the functional NLS elements are harbored within the amino terminal 58 amino acids. While the classical bipartite NLS comprises two clusters of basic amino acids separated by a 10-12 amino acid linker region, exemplified by the NLS of nucleoplasmin [22,23], unconventional bipartite NLSs with extended linker lengths have also been described [24-26]. However, cNLS mapper searches for both conventional and unconventional bipartite NLSs and only detected the former [12]. In addition to monopartite and bipartite NLSs, at least two other classes of NLS have been described: tripartite containing three clusters of basic amino acids similar to those found in L-periaxin and the epidermal growth factor receptor (EGFR) family [27,28], as well as NLSs containing dispersed basic residues within a random coil structure such as that found for 5-lipoxygenase [29]. These NLSs are poorly characterized in comparison with their mono- and bi-partite counterparts and are not predicted by cNLS mapper or PSORT II amino acid prediction algorithms. While the crystal structure of the murine Fanci-Fancd2 heterodimer (ID2) has been solved, the majority of the NLS described in this study was not crystallized precluding speculation about the structure of this region [30]. Protein secondary structure prediction algorithms indicate that this region is comprised largely of random coils. It is also important to note that FANCD2 harbors several putative phosphorylation sites within the amino terminal 58 amino acids (PhosphoSitePlus), which may also contribute to the regulation of its nuclear localization [31]. 

Our studies suggest that FANCD2 is imported to the nucleus *via* an importin α/β-dependent mechanism as treatment with ivermectin, a broad-spectrum inhibitor of importin α/β-dependent nuclear import [13], results in markedly decreased exclusive nuclear localization of D2-NLS-GFP. Furthermore, using mass spectrometry we have recently detected importin β1, as well as the nuclear pore complex proteins NUP160 and NUP155, in FANCD2 immune complexes (Table **S1**). In summary, our functional analyses have revealed the following important points: 1) the NLS is necessary for the nuclear localization of FANCD2, 2) the FANCD2 NLS is required for the nuclear localization of a subset of FANCI, 3) the NLS is necessary for the efficient monoubiquitination of both FANCD2 and FANCI, and 4) the NLS is required for the localization of both FANCD2 and FANCI in chromatin. Consequently, FA-D2 cells expressing FANCD2 NLS deletion mutants are defective in the repair of ICLs. Our studies provide additional important insight into the domain structure of FANCD2, and suggest a novel FANCD2-dependent piggyback mechanism of FANCI nuclear import. Furthermore, our results suggest that a subset of FANCD2 and FANCI are targeted to the nucleus as a heterodimer. These findings lend important insight into the structure and regulation of two poorly characterized tumor suppressor proteins with key early roles in the cellular ICL response.

**Figure 6:**
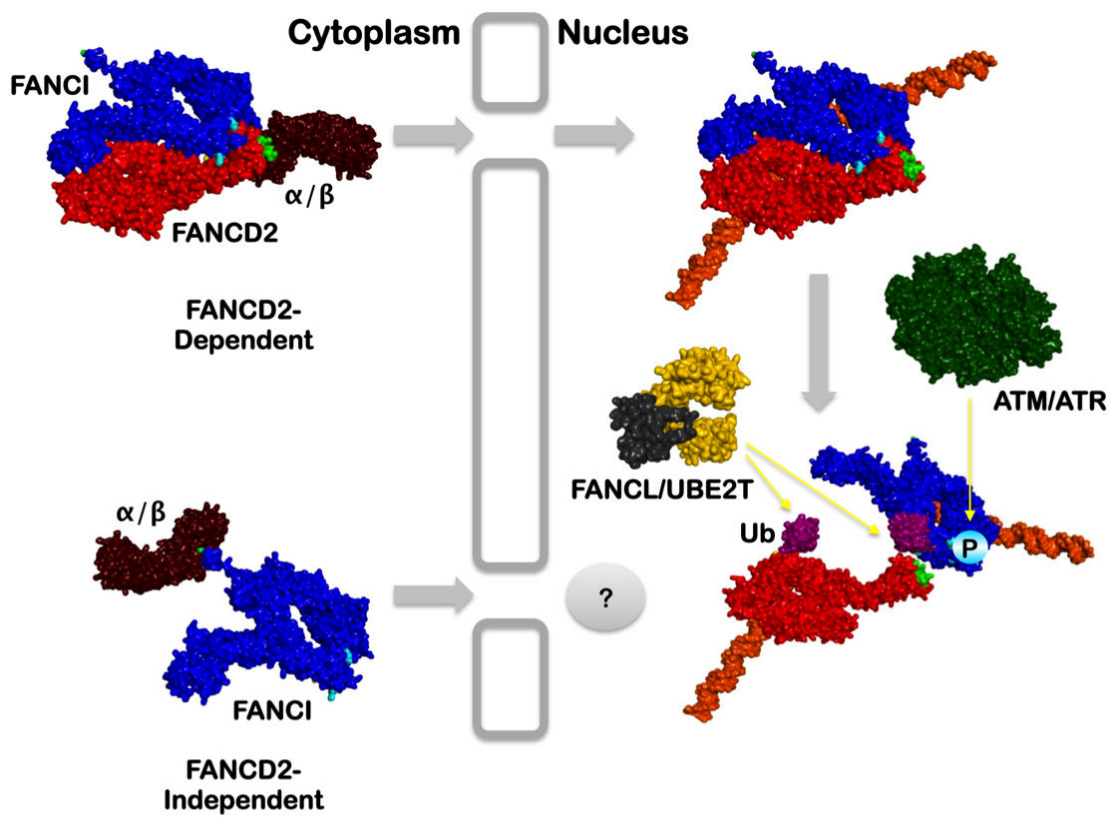
FANCD2-dependent and -independent mechanisms of FANCI nuclear localization. We propose that a subset of FANCI (blue) associates with FANCD2 (red) in the cytoplasm, and that the ID2 heterodimer is transported to the nucleus *via* an importin α/β (brown)-mediated transport mechanism, using the amino terminal FANCD2 NLS (light green). Nuclear ID2 binds to DNA (orange) and is also phosphorylated by the ATM/ATR kinases (dark green). One or both of these events may trigger ID2 complex restructuring, facilitating FANCD2 and FANCI monoubiquitination by FANCL (black), UBE2T (yellow) and the FA core complex (not shown).

Here we have established that FANCI is, at least partially, dependent on FANCD2 for both its nuclear localization and chromatin association: In FA-D2 patient cells, as well as FA-D2 cells expressing the FANCD2 NLS mutants, FANCI localized diffusely to the cytoplasm and nucleus. The introduction of wild type FANCD2 into these cells resulted in a large increase in exclusively nuclear FANCI as well as its chromatin localization, particularly following exposure to MMC. In contrast, we, and others, have observed robust nuclear localization of FANCD2 in FA-I cells, indicating that FANCD2 is not dependent on FANCI for its nuclear localization [32]. A previous study of the patient-derived FANCI R1299X nonsense mutant, which lacks its carboxy-terminal 30 amino acids, demonstrated that FANCI harbors a monopartite NLS in this region [32]. While loss of this NLS reduced FANCI nuclear accumulation, this NLS was not completely necessary for FANCI or FANCD2 nuclear accumulation, strongly suggesting the existence of alternative nuclear import mechanisms for both proteins, consistent with our data [32]. The elucidation of the crystal structure of the ID2 heterodimer indicates that the FANCD2 and FANCI NLSs are spatially separated within this structure [30], arguing against the simultaneous contribution of both NLSs to nuclear import of the ID2 complex. Taken together, these results suggest that FANCI localizes to the nucleus *via* FANCD2-independent and -dependent mechanisms (Figure **6**). These findings are also consistent with the observation that only a minor fraction of the cellular pools of FANCD2 and FANCI physically interact [8,9], reinforcing the concept of ID2 complex-independent functions for both proteins, such as that recently described by Chaudhury and colleagues [33]. A recent study has also established that a fraction of FANCD2 is transported to the nucleus following MMC exposure *via* an indirect interaction with importin 4 (IPO4), which is mediated by the C/EBPδ transcription factor [34]. While clearly important for ICL repair, this mechanism in unlikely to be the major mechanism of FANCD2 nuclear import as robust levels of nuclear FANCD2 were observed in C/EBPδ-null mouse embryonic fibroblasts as well as cells depleted of IPO4 and C/EBPδ [34]. Nevertheless, this C/EBPδ/IPO4-dependent FANCD2 nuclear import mechanism could account for the low levels of nuclear FANCD2-ΔN57 and FANCD2-ΔN57 observed in our studies.

Interestingly, we observed markedly increased MMC-inducible chromosome aberrations and DNA-PK_CS_ pS2056 nuclear foci formation in FA-D2 cells expressing FANCD2-ΔN57, compared to FA-D2 cells expressing LacZ. These results suggest that the FANCD2-ΔN57 mutant may act in a dominant-negative manner. The FA-D2 patient-derived cells used in this study are compound heterozygous for *FANCD2* mutations (*see Materials and Methods*). This variant is detectable by immunoblotting (see Figure **4A** **, top panel**) and is predicted to retain residual or partial function. Indeed, the vast majority of FA-D2 patient-derived cells retain residual FANCD2 function with complete loss of FANCD2 predicted to result in embryonic lethality [15]. Our results suggest that the FANCD2-ΔN57 mutant interferes with residual FANCD2 R1236H function, perhaps competing with FANCD2 R1236H for heterodimerization with FANCI, or in a manner analogous to missense *p53* mutations, by assembling into nonfunctional homo-oligomers, the formation of which has been suggested by previous studies [30,35]. 

Based on our findings, and those of several other groups, we propose the following model for early steps in the FA-BRCA pathway of ICL repair (Figure **6**). A subset of the total cellular pools of FANCD2 and FANCI associate in the cytoplasm to assemble into the ID2 heterodimer. The ID2 heterodimer is transported to the nucleus most likely *via* an importin α/β-mediated transport process, using the amino terminal NLS of FANCD2. Once inside the nucleus the ID2 heterodimer is targeted to sites of ICL damage possibly *via* the association of FANCD2 with PCNA and the replication fork machinery [19]. Recent *in vitro* studies have demonstrated that FANCI binding to DNA is necessary for robust stimulation of the monoubiquitination of FANCD2 [36]. However, analysis of the ID2 crystal structure indicates that the FANCD2 K561 side chain, the site of monoubiquitination, is embedded within the ID2 interface [30]. Furthermore, a solvent accessible tunnel adjacent to FANCD2 K561 is predicted to be too small to accommodate the active site of the UBE2T ubiquitin-conjugating enzyme [30,37]. Therefore, either 1) monoubiquitination occurs on FANCD2 and FANCI monomers prior to ID2 heterodimerization or 2) binding of the ID2 complex to DNA leads to a conformational change in the ID2 structure leading to the exposure of K561R and FANCI K523, and their subsequent monoubiquitination, as has been proposed [36]. A recent study by Sareen and colleagues suggests that activation of the FA-BRCA pathway coincides with dissociation of FANCD2 and FANCI [38]. ID2 dissociation is triggered by ATR/ATM-mediated phosphorylation of a cluster of at least six FANCI SQ motifs, and is followed by the monoubiquitination of FANCD2 [38,39]. Once monoubiquitinated, FANCD2 can then facilitate that recruitment of several structure specific nucleases, including FAN1 and FANCP/SLX4, initiating the process of ICL removal [40-46].

## Materials and Methods

### Cell culture

COS-7, HeLa, and IMR90 cells were grown in Dulbecco's modified Eagle's medium (DMEM) supplemented with 12% v/v FBS, L-glutamine and penicillin/streptomycin. 293FT viral producer cells (Invitrogen) were cultured in DMEM containing 12% v/v FBS, 0.1 mM non-essential amino acids, 1 mM sodium pyruvate, L-glutamine, and penicillin/streptomycin. PD20 FA-D2 (*FANCD2* ^*hy/-*^) cells were purchased from Coriell Cell Repositories (Catalog ID GM16633). These cells harbor a maternally inherited A-G change at nucleotide 376 that leads to the production of a severely truncated protein, and a paternally inherited missense hypomorphic (^hy^) mutation leading to a R1236H change [14]. To generate stable lines expressing wild type or mutant FANCD2, FA-D2 cells were infected with pLenti6.2-FANCD2 (Invitrogen) lentivirus, followed by selection in DMEM supplemented with 12% v/v FBS, L-glutamine, penicillin/streptomycin and 2 μg/ml blasticidin. KEAE FA-D2 cells and KEAE FA-D2 + FANCD2 cells were a kind gift from Detlev Schindler of the University of Würzburg [15]. These cells were telomerase immortalized using pBABE-hTERT and grown in DMEM supplemented with 12% v/v FBS, L-glutamine, penicillin/streptomycin and 0.75 μg/ml puromycin. 

### Antibodies and immunoblotting

For immunoblotting analysis, cell pellets were washed in PBS and lysed in 2% w/v SDS, 50 mM Tris-HCl, 10 mM EDTA. Proteins were resolved on NuPage 3-8% w/v Tris-Acetate or 4-12% w/v Bis-Tris gels (Invitrogen) and transferred to polyvinylidene difluoride (PVDF) membranes. The following antibodies were used: rabbit polyclonal antisera against FANCD2 (NB100-182; Novus Biologicals), FANCI (Dr. Patrick Sung, Yale University), H2A (07-146; Millipore), and mouse monoclonal sera against α-tubulin (MS-581-PO; Neomarkers), GFP (sc-9996; Santa Cruz), and V5 (R96025; Invitrogen).

### Immunofluorescence microscopy

For immunofluorescence microscopy (IF) analysis, cells were seeded in 4-well tissue culture slides (BD Falcon) and treated with mitomycin C (MMC) for 18 h. Soluble cellular proteins were pre-permeabilized with 0.3% v/v Triton X-100 and cells were fixed in 4% w/v paraformaldehyde and 2% w/v sucrose at 4°C followed by permeabilization in 0.3% v/v Triton X-100 in PBS. Fixed cells were blocked for 30 minutes in antibody dilution buffer (5% v/v goat serum, 0.1% v/v NP-40, in PBS) and incubated with primary antibody for 1 h. Cells were washed three times in PBS, as well as permeabilization buffer, and incubated for 30 min at room temperature with an Alexa Fluor 488-conjugated secondary antibody combined with Texas Red labeled phalloidin. The slides were counterstained and mounted in vectashield plus 4’6-diamidine-2-phenylindole dihydrochloride (DAPI) (Vector Laboratories). Nuclear foci were analyzed using a Zeiss AxioImager.A1 upright epifluorescent microscope with AxioVision LE 4.6 image acquisition software. Primary antibodies used for IF were anti-FANCD2 (NB100-182; Novus Biologicals), anti-FANCI (A300-212A; Bethyl Laboratories), anti-DNA-PK_CS_ pS2056 (ab18192; Abcam), and anti-V5 (R960-25; Invitrogen).

### Plasmids, site-directed mutagenesis, and transient transfections

The full length, ΔN57, and ΔN100 *FANCD2* cDNA sequences were TOPO cloned into the pENTR⁄D-TOPO (Invitrogen) entry vector, and subsequently recombined into the pLenti6.2/V5-DEST (Invitrogen) destination vector and used to generate lentivirus for the generation of stable cell lines. The *FANCD2-KRR4NNN* (*FANCD2-3N*) cDNA was generated by site-directed mutagenesis of the wild type *FANCD2* cDNA using the Quikchange Site-directed Mutagenesis Kit (Stratagene). The forward and reverse oligonucleotide sequences used are as follows: FP, 5’-TTCACCATGGTTTCCAACAACAACCTGTCAAAATCTGAGG-3’; RP, 5’-CCTCAGATTTTGACAGGTTGTTGTTGGAAACCATGGTGAA -3’. The FANCD2 GFP fusion vectors D2-1-27-GFP, D2-24-55-GFP, and D2-1-58-GFP were generated by PCR amplifying the coding sequences of amino acids 1-27, 24-55, or 1-58 of FANCD2 and directionally cloning these fragments into the multiple cloning site of pEGFP-N1 (Clontech) (see Methods S1). The FANCI-GFP construct was a gift from Tony Huang in the Department of Biochemistry at New York University School of Medicine. COS-7, HeLa, and IMR90 cells were transiently transfected with plasmid DNA using Fugene 6 or X-tremeGENE 9 (Roche) at a 1:3 ratio (μg DNA:μL Fugene 6) in Opti-MEM. After incubating for 24 h, GFP fluorescence was monitored using a Zeiss AxioImager X-Cite series 120Q inverted fluorescence microscope with AxioVision LE 4.8 image acquisition software. Ivermectin (Sigma) was added to a final concentration of 25 μM 4 h following transfection. 

### Cellular fractionation

Soluble proteins were removed by extraction in cytoskeletal buffer (CSK) (10 mM PIPES pH 6.8, 300 mM sucrose, 100 mM NaCl, 3 mM MgCl_2_, 1 mM EGTA, and 0.5% v/v Triton-X-100) for 10 minutes at 4°C. Pellets were washed once with CSK buffer, lysed in SDS sample buffer (2% w/v SDS, 50 mM Tris-HCl pH 7.4, 10 mM EDTA), boiled for 15 min, followed by sonication for 10 s at 10% amplitude using a Fisher Scientific Model 500 Ultrasonic Dismembrator. 

### Chromosome breakage analysis

Cells were incubated in the absence or presence of MMC for 18 h. Prior to harvesting, cells were treated with 0.1 ug/ml Colcemid (Gibco/Invitrogen) for 2 h; pellets were then incubated in 0.075 M KCl at 37°C for 18 min, followed by fixation in Carnoy’s fixative (3:1 methanol:glacial acetic acid). Cells were dropped onto chilled slides, air-dried, and then stained with 2.5% w/v Giemsa solution (Sigma). Metaphases were analyzed using a Zeiss AxioImager.A1 upright epifluorescent microscope with Axio Vision LE 4.6 image acquisition software.

## Supporting Information

Figure S1 **FANCD2 contains a highly conserved amino-terminal nuclear localization signal, which facilitates nuclear expression of GFP.** (**A**) cNLS mapper (http://nls-mapper.iab.keio.ac.jp/cgi-bin/NLS_Mapper_form.cgi) was used to analyze the FANCD2 amino acid sequence for importin α/β-dependent nuclear localization signals (NLSs), and identified amino acids 2-58 as harboring several putative high scoring bipartite NLSs (**B**). (**C**) A Clustal Omega (http://www.ebi.ac.uk/Tools/msa/clustalo/) multiple sequence alignment of full length FANCD2 corresponding to **Figure 1A**. Hs, *Homo sapiens*; Pt, *Pan troglodytes*; Mm, *Mus musculus*; Cf, Canine *familiaris*; Gg, *Gallus gallus*; Xt, *Xenopus tropicalis*; Dr, *Danio rerio*. (**D**) D2-1-58-GFP localizes primarily to the nucleus. IMR90 cells were transiently transfected with the indicated GFP constructs and analyzed by inverted fluorescence microscopy. (**E** and **F**) HeLa cells were transiently transfected with wild type GFP (GFP-WT) or GFP fused to amino acids 1-58 of FANCD2 (D2-NLS-GFP), incubated in the absence or presence of 25 μM ivermectin for 20 h, followed by analysis by inverted fluorescence microscopy. (**F**) The % of cells exhibiting both cytoplasmic and nuclear (Cyto. + Nucl.) and exclusive nuclear (Nuclear) staining were scored and plotted. Error bars represent the standard errors of the means from two independent experiments. ***, *p* < 0.001. (**G** and **H**) HeLa cells were transiently transfected with the indicated GFP constructs and 24 h later cell pellets were fractionated into soluble (S) and chromatin (C) fractions. Fractions were resolved by SDS-PAGE and immunoblotted with antibodies to GFP, α-tubulin, and H2A. W, unfractionated whole-cell extract. (**H**) The integrated densities of the protein bands from **Figure** **S1G** were quantified using ImageJ image processing and analysis software, and plotted. While the integrated band densities for a single experiment are shown, these experiments were repeated several times with very similar findings. WCE, whole-cell extract.
(PDF)

Figure S2 **The FANCD2 NLS is required for the nuclear localization of a subset of FANCI.** (**A**) KEAE FA-D2 hTERT cells or KEAE FA-D2 hTERT cells stably expressing FANCD2-WT were incubated in the absence (NT) or presence of MMC for 24 h, fixed, stained with rabbit polyclonal anti-FANCD2 or anti-FANCI antibody and counterstained with phalloidin and DAPI. AF-488, Alexa Fluor 488. (**B**) FA-D2 cells stably expressing FANCD2-WT, FANCD2-∆N57, or FANCD2-∆N100 were incubated in the absence (NT) or presence of MMC for 24 h, fixed, stained with mouse monoclonal anti-V5 (red) to detect V5-tagged FANCD2 and rabbit polyclonal anti-FANCI (green) and counterstained with DAPI (blue). IF microscopy was performed with (+ Pre-Perm) and without (No Pre-Perm) a pre-permeabilization step (*see Materials and Methods*). The pre-permeabilization step leads to complete loss of fluorescent signal for FANCD2-ΔN57 and FANCD2-ΔN100 because of the high solubility of these proteins (see **Figure 2B**), while this step is necessary for the resolution of FANCI fluorescence signal.
(PDF)

Figure S3 **The FANCD2 NLS is required for efficient FANCI chromatin association.** FA-D2 cells stably expressing FANCD2-WT (WT), FANCD2-∆N57 (∆N57), FANCD2-∆N100 (∆N100) or FANCD2-3N (3N) were incubated in the absence or presence of MMC for 18 h and cell pellets were fractionated into soluble and chromatin-associated fractions (see **Figures 4B and C**). The total integrated densities of the chromatin-associated (C) nonubiquitinated and monoubiquitinated FANCI protein bands were quantified using ImageJ image processing and analysis software, and plotted. While the integrated band densities for a single experiment are shown, these experiments were repeated several times with similar findings. NT, not treated; MMC, MMC-treated.
(TIF)

Figure S4 **The FANCD2 NLS deletion mutants fail to rescue the MMC sensitivity of FA-D2 cells.** FA-D2 cells stably expressing FANCD2-WT, FANCD2-K561R, FANCD2-∆N57, FANCD2-∆N100, or FANCD2-3N were treated with the indicated concentrations of MMC for 7-10 days. Cells were fixed and stained with crystal violet and percent survival calculated and plotted. Each measurement was performed in triplicate and experiments were performed multiple times with similar results. The 20% trimmed means (_20%_) for all recorded measurements were calculated and plotted. Error bars represent the standard errors of the means.
(TIF)

Table S1 **Detection of importin subunit β1, NUP160 and NUP155 in FANCD2 immune complexes.** FANCD2 immune complexes were analyzed using a LTQ Orbitrap Velos hybrid mass spectrometer.
(TIF)

Methods S1 (DOCX)
