# Supplementary material for: Coordinate Nuclear Targeting of the FANCD2 and FANCI Proteins via a FANCD2 Nuclear Localization Signal
Source: PLoS One. 2013 Nov 21;8(11):e81387. doi: 10.1371/journal.pone.0081387 (PMC3836817; doi:10.1371/journal.pone.0081387)
Supplement: Figure S1 — FANCD2 contains a highly conserved amino-terminal nuclear localization signal, which facilitates nuclear expression of GFP. (A) cNLS mapper (http://nls-mapper.iab.keio.ac.jp/cgi-bin/NLS_Mapper_form.cgi) was used to analyze the FANCD2 amino acid sequence for importin α/β-dependent nuclear localization signals (NLSs), and identified amino acids 2-58 as harboring several putative high scoring bipartite NLSs (B). (C) A Clustal Omega (http://www.ebi.ac.uk/Tools/msa/clustalo/) multiple sequence alignment of full length FANCD2 corresponding to Figure 1A. Hs, Homo sapiens; Pt, Pan troglodytes; Mm, Mus musculus; Cf, Canine familiaris; Gg, Gallus gallus; Xt, Xenopus tropicalis; Dr, Danio rerio. (D) D2-1-58-GFP localizes primarily to the nucleus. IMR90 cells were transiently transfected with the indicated GFP constructs and analyzed by inverted fluorescence microscopy. (E and F) HeLa cells were transiently transfected with wild type GFP (GFP-WT) or GFP fused to amino acids 1-58 of FANCD2 (D2-NLS-GFP), incubated in the absence or presence of 25 μM ivermectin for 20 h, followed by analysis by inverted fluorescence microscopy. (F) The % of cells exhibiting both cytoplasmic and nuclear (Cyto. + Nucl.) and exclusive nuclear (Nuclear) staining were scored and plotted. Error bars represent the standard errors of the means from two independent experiments. ***, p < 0.001. (G and H) HeLa cells were transiently transfected with the indicated GFP constructs and 24 h later cell pellets were fractionated into soluble (S) and chromatin (C) fractions. Fractions were resolved by SDS-PAGE and immunoblotted with antibodies to GFP, α-tubulin, and H2A. W, unfractionated whole-cell extract. (H) The integrated densities of the protein bands from Figure S1G were quantified using ImageJ image processing and analysis software, and plotted. While the integrated band densities for a single experiment are shown, these experiments were repeated several times with very similar findings. WCE, whole-cell ext [file pone.0081387.s001.pdf]

A

Predicted NLSs in query sequence

```
MVSKRRLSKSEDKESLTEDASKTRKQPLSKKTKKSHIANEVEENDSIFVK  50
LLKISGIILKTGESQNQLAVDQIAFQKKLFQTLRRHPSYPKIIEEFVSGL  100
ESYIEDEDSFRNCLLSCERLQDEEASMGASYSKSLIKLLLGIDILQPAII  150
KTLFEKLPEYFFENKNSDEINIPRLIVSQLKWLDRVVDGKDLTTKIMQLI  200
SIAPENLQHDIITSLPEILGDSQHADVGKELSDLLIENTSLTVPILDVLS  250
SLRLDPNFLLKVRQLVMDKLSSIRLEDLPVIIKFILHSVTAMDTLEVISE  300
LREKLDLQHCVLPSRLQASQVKLKSKGRASSSGNQESSGQSCIILLFDVI  350
KSAIRYEKTISEAWIKAIENTASVSEHKVFDLVMLFIIYSTNTQTKKYID  400
RVLRNKIRSGCIQEQLLQSTFSVHYLVLKDMCSSILSLAQSLLHSLDQSI  450
ISFGSLLYKYAFKFFDTYCQQEVVGALVTHICSGNEAEVDTALDVLLELV  500
VLNPSAMMMNAVFVKGILDYLDNISPQQIRKLFYVLSTLAFSKQNEASSH  550
IQDDMHLVIRKQLSSTVFKYKLIGIIGAVTMAGIMAADRSESPSLTQERA  600
NLSDEQCTQVTSLLQLVHSCSEQSPQASALYYDEFANLIQHEKLDPKALE  650
WVGHTICNDFQDAFVVDSCVVPEGDFPFPVKALYGLEEYDTQDGIAINLL  700
PLLFSQDFAKDGGPVTSQESGQKLVSPLCLAPYFRLLRLCVERQHNGNLE  750
EIDGLLDCPIFLTDLEPGEKLESMSAKERSFMCSLIFLTLNWFREIVNAF  800
CQETSPEMKGKVLTRLKHIVELQIILEKYLAVTPDYVPPLGNFDVETLDI  850
TPHTVTAISAKIRKKGKIERKQKTDGSKTSSSDTLSEEKNSECDPTPSHR  900
GQLNKEFTGKEEKTSLLLHNSHAFFRELDIEVFSILHCGLVTKFILDTEM  950
HTEATEVVQLGPPELLFLLEDLSQKLESMLTPPIARRVPFLKNKGSRNIG  1000
FSHLQQRSAQEIVHCVFQLLTPMCNHLENIHNYFQCLAAENHGVVDGPGV  1050
KVQEYHIMSSCYQRLLQIFHGLFAWSGFSQPENQNLLYSALHVLSSRLKQ  1100
GEHSQPLEELLSQSVHYLQNFHQSIPSFQCALYLIRLLMVILEKSTASAQ  1150
NKEKIASLARQFLCRVWPSGDKEKSNISNDQLHALLCIYLEHTESILKAI  1200
EEIAGVGVPELINSPKDASSSTFPTLTRHTFVVFFRVMMAELEKTVKKIE  1250
PGTAADSQQIHEEKLLYWNMAVRDFSILINLIKVFDSHPVLHVCLKYGRL  1300
FVEAFLKQCMPLLDFSFRKHREDVLSLLETFQLDTRLLHHLCGHSKIHQD  1350
TRLTQHVPLLKKTLELLVCRVKAMLTLNNCREAFWLGNLKNRDLQGEEIK  1400
SQNSQESTADESEDDMSSQASKSKATEDGEEDEVSAGEKEQDSDESYDDS  1450
D                                                   1451
```

B

Position	Sequence	Score
2-27	VSKRRLSKSEDKESLTEDASKTRKQP	10.1
24-55	RKQPLSKKTKKSHIANEVEENDSIFVKLLKIS	7.2
2-30	VSKRRLSKSEDKESLTEDASKTRKQPLSK	6.8
24-58	RKQPLSKKTKKSHIANEVEENDSIFVKLLKISGII	5.7
30-55	KKTKKSHIANEVEENDSIFVKLLKIS	5.6

Figures S1A and B

```
Hs    1 -MVSKRRLSKSEDKES-LTEDA-SKTRKQPLSKKTKKSHIANEVEENDSIFVKLLKISGI
Pt    1 -MVSKRRLSKSEDKES-LTEDA-SKTRKQPLSKKTKKSHIANEVEENDSIFVKLLKVSGI
Mm    1 -MISKRRRLDSEDKEN-LTEDA-SKT--MPLSKLAKKSHNSHEVEENGSVFVKLLKASGL
Cf    1 -MVSKRRLSKSEDKES-LTEDA-SKARKQPLSKKTKKSHVRVEVEENDSVFVKLLKTSGV
Gg    1 -MVSKRKLSKIDAAEESSKTDLQSRCPETKRSRISDKRAPSQGGLENEGVFEELLRTSGI
Xt    1 -MVAKRRLSRSDDREESFAADTS-KNKKCRTSSKKTKALPQDGVVENDSIFVQLLKSSGM
Dr    1 MMRKKKRSSVTVD--EDPTLDVS-KAKKSKSSGRSTKSSVQD--SCQDSVFVQFLKESGV

Hs   58 ILKTGESQNQLAVDQIAFQKKLFQTLRRHPSYPKIIEEFVSGLESYIEDEDSFRNCLLSC
Pt   58 ILKTGESQNQLAVDQIAFQKKLFQTLRRHPSYPKIIEEFVSGLESYIEDEDSFRNCLLSC
Mm   56 TLKTGENQNQLGVDQVIFQRKLFQALRKHPAYPKVIEEFVNGLESYTEDSESLRNCLLSC
Cf   58 ILKTGESQNQLTVDQIVFQKKLFQTLRKHPSYPKIIEEFVSGLESYIEDQDSFRNCLLSC
Gg   60 ILKVGEGQNEIAVDQTAFQKKLRVALEKHPSYPGVVNEFISGLESHIKDRSQFKNCLLPC
Xt   59 TLKSGDRQNEIAVDQAVFQKKLHQALRKHPRHPNVIQEFISGLESHIEDRDTFRNCLLPC
Dr   56 TLTPGSTANEIAVDQVIFQKRLQQRLRKSPRFPNIIQEFISALETYIEDPERFRNCLLPC

Hs  118 ERLQDEEA-SMGASYSKSLIKLLLGIDILQPAIIKTLFEKLPEYFFENKNSDEINIPRLI
Pt  118 ERLQDEEA-SMGASYSKSLIKLLLGIDILQPAIIKTLFEKLPEYFFENKNSDEINIPRLI
Mm  116 ERLQDEEA-SMGTFYSKSLIKLLLGIDILQPAIIKMLFEKVPQFLFESENRDGINMARLI
Cf  118 ERLQDEEA-SMGTSYSKSLIKLLLGIDILQPAIIKTLFEKLPEFLFENVNSDGLNIPRLI
Gg  120 TPARTEGSRTLVHSYCESLIKLLLGIKILQPAVVTLLLEKIPEFFFDVVGTFGTNFPRLI
Xt  119 GNRQETEASTMVGSFHDSLMKLLLGLEILQESVINTLFEKLPEFLYDSVGSDGISIPRLI
Dr  116 VPAPTSQQDTNSSSYQESLVRLLLGIEMLQTLVINTLFEKLPEFMFEGASEDGLNVPRLI

Hs  177 VSQLKWLDRVVDGKDLTTKIMQLISIAPENLQHDIITSLPEILGDSQHADVGKELSDLLI
Pt  177 VSQLKWLDRVVDGKDLTTKIMQLISIAPENLQHDIITSLPEILGDSQHADVGKELSDLLI
Mm  175 INQLKWLDRIVDGKDLTAQMMQLISVAPVNLQHDFITSLPEILGDSQHANVGKELGELLV
Cf  177 ISQLKWLDRIVDGKDLTTKIMQLISIAPLYLQHDFVTSLPEILGDSQHADVGKELSELLM
Gg  180 VNQFKWLDGLLDSQDLVKKLMQMLSVSPVPIQHDIITSLPEILEDSQQNEVAREISCLLK
Xt  179 INQFKWLDRILDCKDLTLKIMQLISVAPVDIQHDIITSLPEILEDSQHNDVARELNSLLQ
Dr  176 VNQLKWLDRIVDGKDLSSKLMQMVSVAPVEIQRDIITSLPEILEDSQHGDMAKELNGLLQ

Hs  237 ENTSLTVPILDVLSSLRLDPNFLLKVRQLVMDKLSSIRLEDLPVIIKFILHSVTAMDTLE
Pt  237 ENTSLTVPILDVLSSLRLDPNFLLKVRQLVMDKLSSIRLEDLPVIIKFILHSITAVDALE
Mm  235 QNTSLTVPILDVFSSLRLDPNFLSKIRQLVMGKLSSVRLEDFPVFVKFLLHSVTDTTSLE
Cf  237 ENTLLTVPILDVLSSLRLDPKLLTQVRQLVMSKLLSVKLDDLPVIIKFILHSVTATDALE
Gg  240 QGRRLTVPILDALSRLDLDAELLAKVRQSAMTIVPSVKLEDLPVVIKFILHNVKAADAVE
Xt  239 QNTQLTVPILDALSSLNINTDLLSEVRQSVMSTLSAVELEDLPVIIKFILHAVTPSDAVE
Dr  236 QNTQLAVPILDALSSLNLSSTLLSEVREAVMGTLSAVQLEDLPVVVKFVLHSISASDANE

Hs  297 VISELREKLDLQHCVLPSRLQASQVKLKSKGRASS-SGNQESSGQSCIILLFDVIKSAIR
Pt  297 VISELREKLDLQHCVLPSRLQASQVKLKSKGRASS-SGNQESSGQSCIILLFDVIKSAIR
Mm  295 VIAELRENLNVQQFILPSRIQASQSKLKSKGLASS-SGNQENSDKDCIVLVFDVIKSAIR
Cf  297 VISELREKLDLQHCILPSRLQASQSKLKNKGRQSS-SGNQENSGQDCVILLFDVIKSAVR
Gg  300 VISDLRKSLDLSSCVLPLQLLGSQRKLKSQAQASS-SMSQVTTSQNCVKLLFDVIKLAVR
Xt  299 VISELRKKLDLESCSSLAQIHATQNKERNKPQAGSS--VNKTKSSDCVSLMMDVIKSAVR
Dr  296 VVCDLRKKLELEQCVLPAVLQASQSRMKNKTVSRSSSVPSCSSGQDSVALILDVIKSAIR

Hs  356 YEKTISEAWIKAIENTASVSEHKVFDLVMLFIIYSTNTQ-TKKYIDRVLRNKIRSGCIQE
Pt  356 YEKTISEAWIKAIENTASVSEHKVFDLVMLFIIYSTNTQ-TKKYIERVLRNKIRSGCIQE
Mm  354 YEKTISEAWFKAIERIESAAEHKALDVVMLLIIYSTSTQ-TKKGVEKLLRNKIQSDCIQE
Cf  356 YEKIISEAWIKAIESTASVSEHKTFDLAMLLIIYSTNTQ-TKKYIERVLRNKIRSGCFQE
Gg  359 FQKDVSEAWIKAIENSTSVSDHKVLDLIVLLLIHSTNSK-NRKQTEKVLRSKIRLGCMPE
Xt  357 FQKHMSEAWIKAIENVDTVGDHKVSDLIVLLILYTTQTNSSKKQAERVLRNKIRSGFISD
Dr  356 FQKTISEAWLKAIENIDESEDHKVVDLLVLFILHSTNANHSRRGAERVLKVKVRKGLIQE

Hs  415 QLLQSTFSVHYLVLKDMCSSILSLAQSLLHSLDQSIISFGSLLYKYAFKFFDTYCQQEVV
Pt  415 QLLQSTFSVHYLVLKDMCSSILSLAQSLLHSLDQSIISFGSLLYKYAFKFFDTYCQQEVV
Mm  413 QLLDSAFSTHYLVLKDICPSILLLAQTLFHSQDQRIILFGSLLYKYAFKFFDTYCQQEVV
Cf  415 ELLQSTFYIHYLVLKDICPSILSLAQSLLHSLDQSIILFGSLLYKYSFKFFDTYCQQEVV
Gg  418 QLMQNAFQNHSMVIKDFFPSILSLAQTFLHSAHPAVVSFGSCMYKQAFAVFDSYCQQEVV
Xt  417 QLLQNAFRNHSQVLRDYFPSILSLAQSMLRSAEQSVVSFGSLMYKSAFSVFDSYCQQEVV
Dr  416 SLLQKTFKGHAQVMRGYFPSILALAQGLLRSPDCCVVPFGGHMYKQAFTAFDSYCQQEVV
```

```
Hs  475  GALVTHICSGNEAEVDTALDVLLELVVLNPSAMMMNAVFVKGILDYLDNISPQQIRKLFY
Pt  475  GALVTHICSGNEAEVDTALDVLLELVVLNPSAMMMNAVFVKGILDYLDNISPQQIRKLFY
Mm  473  GALVTHVCSGTEAEVDTALDVLLELIVLNASAMRLNAAFVKGILDYLENMSPQQIRKIFC
Cf  475  GALVTHICSGSEAEVDTALDALLELVVLNPSAMRLNAVFVKGILDYLDNMSPQQIRKLFY
Gg  478  CALVTHVCSGNETELDISLDVLTDLVILHPSLLLRYATFVKTILDSMQKLNPCQIRKLFY
Xt  477  GALVTHVCSGYPAEVDVSLDVLTDLVSSHAAAVALYAVFVKGILDYLDNLNAQQIRKLFH
Dr  476  GSLVTHACSGVSGEVDVALELLCELVSQKPAEMSQFTVFVKGILDYMDNLTSQQIRRLFH

Hs  535  VLSTLAFSKQNEASSHIQDDMHLVIRKQLSSTVFKYKLIGIIGAVTMAGIMAADRSESPS
Pt  535  VLSTLAFSKQNEASSHIQDDMHLVIRKQLSSTVFKYKLIGIIGAVTMAGIMAADRSESPS
Mm  533  ILSTLAFSQQPGTSNHIQDDMHLVIRKQLSSTVFKYKLIGIIGAVTMAGIMAEDRSVPSN
Cf  535  ILSSLAFSKH-EASSHIQDDMHLVIRKQLSSTVFKYKLIGIIGAVTMAGIMAADRSS--N
Gg  538  ILSTLAFSQRQ-EGSYIQDDMHMVIRKWLSSSVPNHKQMGIIGAVTMMGSVALKRNEADG
Xt  537  ILSVLAFSRGQ-EGSHIQDDMFIVIRKQLSSTALKYKRIGIIGAVRMVGSMAVNNNVTKH
Dr  536  LLSRLAFGQEQ-HGGHIQDDMHIVIRKQLSSTVPKYKRIGIIGAVMMVGSMGACRNKHD-

Hs  595  L--TQERANLSDEQCTQVTSLLQLVHSCSEQSPQASALYYDEFANLIQH--EKLDPKALE
Pt  595  L--TQERANLSDEQCTQVTSLLQLVHSCSEQSPQASALYYDEFANLIQH--EKLDPKPWE
Mm  593  S--SQRSANVSSEQRTQVTSLLQLVHSCTEHSPWASSLYYDEFANLIQE--RKLAPKTLE
Cf  592  L--TQGRTDLSNEEYTQVTSLLQLVHSCSEQSPQASALYYDEFANLIQG--GKLAPKALE
Gg  597  GLL--ERPELSIECDGQLSTLLDLVGFCCEQTPEVLALYYDELANLIEKQKGNLDLQLLD
Xt  596  GSKNPENNPLSAESFRQVTALLELVQTCSEQVPEASALYYDELSSLVQK--RNLDPLVMS
Dr  594  ---GSQGGTLPKETNRQVMALLELVRSCSESSPEAAALCYDELANLLQT--CKLDPLVQA

Hs  651  WVGHTICNDFQDAFVVDSCVVPEGDFPFPVKALYGLEEYDTQDGIAINLLPLLFSQDFAK
Pt  651  WVGHTICNDFQDAFVVDSCVVPEGDFPFPVKALYGLEEYDTQDGIAINLLPLLFSQDFAK
Mm  649  WVGQTIFNDFQDAFVVDFCAAPEGDFPFPVKALYGLEEYSTQDGIVINLLPLFY-QECAK
Cf  648  WVGQTILNDFQDAFVVDFCVSPEGDYPFPVKALYGLEEYSSHDGIVINLLPLMFSQDFAK
Gg  655  KFGKSLVEDFPNDFVVDLSPTVDGSFLFPVKSLYNLDEDETQGAIAINLLPLVSQSEPGR
Xt  654  WVGKTVLTDFQDDFVDDLTPTVEGNYIFPVKAMYNLDEDDSQGGIAINILPVLSKDMCSR
Dr  649  WIGKSVLEDFQDDFVVDLGPDISGAFMFPTSVLYNLDEEESEGGIAVNLLPLMALDQQHK

Hs  711  DGGPVTSQESGQKLVSPLCLAPYFRLLRLCVERQHNGNLEEIDGLLDCPIFLTDLEPGEK
Pt  711  DGGPVTSQESGQKLVSPLCLAPYFRLLRLCVERQHNGNLEEIDGLLDCPIFLTDLEPGEK
Mm  708  DASRATSQESSQRSMSSLCLASHFRLLRLCVARQHDGNLDEIDGLLDCPLFLPDLEPGEK
Cf  708  DGGRMTSKESDQKVVSPLCLAPYFRLLRLCVERQHDGNLEEIDGLLDCPIFLTDLEPGER
Gg  715  VAD--EMSNSRKRVVSPICLSPCFRLLRLYTGEQNNGSLEEIDALLGCPLYLTDLEVEGK
Xt  714  -GAEQAANKEESRVVSPICLSPFFRLLRLCIEDQHEGNLEEIDALLGCPLYLTDLEITEK
Dr  709  TDTSQPAGVKRERRVSPLCLSPFFRLLRLCEEVQHEGDLEEIDALLGCPLILTDMEVVEK

Hs  771  LESMSAKERSFMCSLIFLTLNWFREIVNAFCQETSPEMKGKVLTRLKHIVELQIILEKYL
Pt  771  LESMSAKERSFMCSLIFLTLNWFREIVNAFCQETSPEMKGKVLTRLKHIVELQIILEKYL
Mm  768  LESMSAKDRSLMCSLTFLTFNWFREVVNAFCQQTSPEMKGKVLSRLKDIVELQGILEKYL
Cf  768  LESMSVKEHSFMCSLIFLTLNWFREVVNAFCQQTSPEMKGKVLTRLKHIVELQRILEKYL
Gg  773  LDSLSKQEREFLCSLLFYALNWFREVVNAFCQQQDAEMKGKVLTRLQNITELQNVLGKCL
Xt  773  MESLSKQEREFLCSLLFFALNWFREIVNAFCKQQDLDMKSKVLTRLQNITWLQSVLEKCL
Dr  769  VDSLSKSEREFLCSLLFHTINWFREVVNAFCRQNDPEMKMKVVTRIQNITYLQSLLQTCL

Hs  831  AVTPDYVPPLGNFDVETLDITPHTVTAISAKIRKKGKIE-RKQKTDGSKTSSSDTLSEEK
Pt  831  AVTPDYVPPLGNFDVETLDITPHTVTAISAKIRKKGKIE-RKQKTDGSKTSSSDTLSEEK
Mm  828  AVIPDYVPPFASVDLDTLDMMPRSSSAVAAKNRNKGKTGGKKQKADSNKASCSDTLLTED
Cf  828  AVTPDYVPPLANFDLETLDVTPPTTAAISAKIRKQGKIG-KKRKADGSKTSSPDTLSKED
Gg  833  AATPGYVPPPATFDSEAPEGVPSINAGGPVRKKNG-----KKRKSDSSKACSAERTQADE
Xt  833  AASPGYIPPSAQFDSEPQEVLPSAIASAPAKKAKKGQAGCKTQKSAASKNSSADSSQLEE
Dr  829  AATPGYIPPQANFDGESADVMIPSSSVVQQKKGKKESSGR-KRKASASKNSSGDKSQLEG

Hs  890  NSECDPTPSHRGQLNKEFT----GKEEKTSLLLHNSHAFFRELDIEVFSILHCGLVTKFI
Pt  890  NSECDPTPSHRGQLNKEFT----GKEEKTSLLLHNSHAFFRELDIEVFSILHCGLVTKFI
Mm  888  TSECDMAPSGRSHVDKEST----GKEGKTFVSLQNYRAFFRELDIEVFSILHSGLVTKFI
Cf  887  SSECDPTPSNRSQLEKEFK----GKEERTSVSLQNYHAFFRELDIEVFSILHCGLVTKFI
Gg  888  SSDGNQPDT---ELSELEKSAAEKETGNPLAQLQSYRPYFRELDLEVFSVLHCGLLTKSI
Xt  893  HQDTEKSELEKTQPEKFSTGREKEESSKPTINLNSYRAYFRELDLEVFTVLQCGLLTRSV
Dr  888  AADAEESQP---DPPEKESEKEKEKDGKSGVSLSSYWPFFRELDVEALSVLQCGLLSRTL
```

```
Hs  946 LDTEMHTEATEVVQLGPPELLFLLEDLSQKLESMLTPPIARRVPFLKNKGSRNIGFSHLQ
Pt  946 LDTEMHTEATEVVQLGPPELLFLLEDLSQKLESMLTPPIARRVPFLKNKGSRNIGFSHLQ
Mm  944 LDTEMHTEATEVVQLGPAELLFLLEDLSQKLENMLTAPFAKRICCFKNKGRQNIGFSHLH
Cf  943 LDTEMHTEATEVVQLQPPELLFLLEDLSQKLENMLTPSVAKRIPFLKSKGNRNIGFSHLH
Gg  945 LDTEMHTEASEVVQLGPAELCFLLDDMCWKLEHVLTPGSTRRVPFLKERGNKDVGFSHLC
Xt  953 LDSEMHTKATEVVQLGPAELVFLLEDLFRKTEHMLTST-VKRVMFLKVKGSRNIGFSQLR
Dr  945 LDTELHSKLREEVQLGPAELLFLLEDMWRKLDFSLTSAPAKKAPFLKGKMDKAVGFAHLQ

Hs 1006 QRSAQEIVHCVFQLLTPMCNHLENIHNYFQCLAAENHGVVDGPGVKVQEYHIMSSCYQRL
Pt 1006 QRSAQEIVHCVFQLLTPMCNHLENIHNYFQCLAAENHGVVDGPAVKVQEYHIMSSCYQRL
Mm 1004 QRSVQDIVHCVVQLLTPMCNHLENIHNFFQCLGAEHLSADDKARATAQEQHTMACCYQKL
Cf 1003 QRSAQEVAHSIVQLLTPMCNHLENIHNYFQCLAAENQGVVDGPRTKVEEYHMMSSCYQRL
Gg 1005 QRSPKEVAVCVVKLLKPLCNHMENMHNYFQTVI-PNQGVVDESGLNIQEYQLMSSCYHQL
Xt 1012 QKTPQEVAQAVIHLLNSLCNHTENMHNYFQTLMLENHGVVDAPSVDIKEHQYMSTCYQLL
Dr 1005 QKNAKEIATCCSDLLPTLCTHLENCHNHFQTLLTENQGVVDAPNVDVKENQLMCSAYQLL

Hs 1066 LQIFHGLFAWSGFSQPENQNLLYSALHVLSSRLKQGEHS-QPLEELLSQSVHYLQNFHQS
Pt 1066 LQIFHGLFAWSGFSQPENQNLLYSALHVLSSRLKQGEHS-QPLEELLSQSFHYLQNFHQS
Mm 1064 LQVLHALFAWKGFTHQSKHRLLHSALEVLSNRLKQMEQD-QPLEELVSQSFSYLQNFHHS
Cf 1063 LQIFHGLFAWNGFFQLENYNLLYSALEVLTNRLKQGEPD-QSLDELLSQSFNYLQNFSHS
Gg 1064 LLAFRLLFAWSGFSQHENSNLLRSALQVLADRLKPGETEFLPLEELISESFQYLLNFQAS
Xt 1072 LQVFHTLFCWSGFSQHENRKLLKSALIALAGRLKETEAE-PALDELVRQSFNYLKNLHSS
Dr 1065 LQVLHTMFSWSGFSIPEHRGLLKSALGILAGRLKEDQAD-LTLDQLSRHAFEYLQNFRST

Hs 1125 IPSFQCALYLIRLLMVILEKSTASA--QNKEKIASLARQFLCRVW--PSGDKEKSNISND
Pt 1125 IPSFQCALYLIRLLMVILEKSTAST--QNKEKIASLARQFLCRVW--PSGDKEKSNISND
Mm 1123 VPSFQCGLYLLRLLMALLEKSAVPN--QKKEKLASLAKQLLCRAW--PHGEKEKNPTFND
Cf 1122 IPNFQCALYLIRLLMVILEKSTAPT--QKKEKIASLAKQFLCRVW--PSGEKEKSSIPTE
Gg 1124 IPSFQCAFILTQVLMAISEKPMT---GWKREKMASLAKQFLCQSWMKPGGDREKGSHFNS
Xt 1131 VPTCSSALCLTQLLIVIAEKANVLH--YREQETASMAKQFLCQPWIQPSGEREKGTRYHE
Dr 1124 VPSLNTALCLTQLLIVLPQYGGSNHRTYRE-QITSLTKHFLCQEWVTASGEKERGNKYNE

Hs 1181 QLHALLCIYLEHTESILKAIEEIAGVGVPELINSPKDASSSTFPTLTRHTFVVFFRVMMA
Pt 1181 QLHALLCIYLEHTDSILKAIEEIAGVGVPELINSPKDASSSTFPTLTRHTFVVFFRVMMA
Mm 1179 HLHDVLYIYLEHTDNVLKAIEEITGVGVPELVSAPKDAASSTFPTLTRHTFVIFFRVMMA
Cf 1178 QLHTLLSVYLEHTDSVLKAIEEIAGVGVPELINSPKDASSSMFPTLTRQSFVIFFRVMMA
Gg 1181 ALHTLLCVYLEHTDNILKAIEEISSVGVPELINSAKDGCSSTYPTLSRQTFPVFFRVMMA
Xt 1189 NLQSLLCIYLENTDDVLKAVEEIAGVGVPELVNAAKDAGSSSYPTLTRQTFIVFFRVMMD
Dr 1183 TLQTLLSIYLEHTDDVLKAVEEIAGEGVPELLNAAKDANSRSWPTLNRQTFLVYYKCMMS

Hs 1241 ELEKTVKKIEPGTAADSQQIHEEKLLYWNMAVRDFSILINLIKVFDSHPVLHVCLKYGRL
Pt 1241 ELEKTVKKIEPGTAADSQQIHEEKLLYWNMAVRDFSILINLIKVFDSHPVLHVCLKYGRL
Mm 1239 ELEKTVKGLQAGTAADSQQVHEEKLLYWNMAVRDFSILLNLMKVFDSYPVLHVCLKYGRR
Cf 1238 ELEKTVKGLQAGTAADSQQIHEEKLLYWNMAVRDFSILINLIKVFDSRPVLHVCLKYGRL
Gg 1241 QLESSVKSIPAGKPSDSGEVQLEKLLKWNIAVRNFHILINLVKVFDSRPVLSICLKYGRL
Xt 1249 KLEKCVKCIPSGKKAETLQEQTEQLLRWNLAVRDFHILVNLVKVFDSRPVLSICLKYGRM
Dr 1243 ELEKAVRKIPPSKQMDNQEMQSEKLLTRNLAVRDFHILINLVKMFDSRPVLTVCLKYGRL

Hs 1301 FVEAFLKQCMPLLDFSFRKHREDVLSLLETFQLDTRLLHHLCGHSKIHQDTRLTQHVPLL
Pt 1301 FVEAFLKQCMPLLDFSFRKHREDVLSLLETFQLDTRLLHHLCGHSKIHQDTRLTQHVPLL
Mm 1299 FVEAFLKQCMPLLDFSFRKHREDVLSLLQTLQLNTRLLHHLCGHSKIRQDTRLTKHVPLL
Cf 1298 FVEAFLKQCMPLLDFSFRKHREDVLSLLETFQLNTRLLHHLCGHSKIHQDTKLTKHVPLL
Gg 1301 FVEAFLKLAMPLLDHSFKKHRDDVQSLLKTLQLSTRQLHHMCGHSKIHQDLGLTNHVPLL
Xt 1309 FVETFLKLGMPLLDCCFKKHREDVQSLLKTLQLSTRQLHHMCGHSKIHQDTALTNHVPLL
Dr 1303 FLESFLKLGMPLLDYSFKKHKEDVQGLLKTFQLSTRQLHHMCGHSKIRQDTGLTNHVPAL

Hs 1361 KKTLELLVCRVKAMLTLNNCREAFWLGNLKNRDLQGEEIKSQNSQ--ESTADESEDDMSS
Pt 1361 KKTLELLVCRVKAMLTLNNCREAFWLGNLKNRDLQGEEIKSQNSQ--ESTADESEDDMSS
Mm 1359 KKSLELLVCRVKAMLVLNNCREAFWLGTLKNRDLQGEEIISQDPSSSESNAEDSEDGVTS
Cf 1358 KKTLELLVCRVKAMLIFNNCREAFWLGNLKNRDLQGEEIISQNSQ--ESAAEESEDDTAS
Gg 1361 KKSLEQFVYRVKAMLAFNHCQEAFWVGVLKNRDLQGEEILSQASAAPEEDSAEGSEEDTE
Xt 1369 KKTLELFVYRVKAMLVLNNCQDAFWLGNLKNRDLQGEEILSQVSQDSEGEEEQESQLPPE
Dr 1363 KKSLEQFVYRVKAMLTLNHCQEAFWLGNLKNRDLKGEEILSQRSQAADEEEEESSQLQSE
```

```
Hs 1419 Q-ASKSKATEDGEEDEVSAGEKEQDSD--ESYDDSD------------------
Pt 1419 Q-ASKSKATEVSLQNPPESGIDGCILLIVLSWWSRTLPTYVYCQMLLCPFPFPP
Mm 1419 H-VSRNRATEDGEDEAS-DEQKDQDSDESDDSSS--------------------
Cf 1416 Q-VSKSKTTEDAENDEVSDGEKDQDSDESEDSD---------------------
Gg 1421 D---SAAEEPDGTDSDS-GGAGR-------------------------------
Xt 1429 IEETEEEEENEGSDKEI-EGEDEHN----EDEDS--------------------
Dr 1423 AEESDSDERNDGQEEVE-ESDESD------------------------------
```

Figure S1C

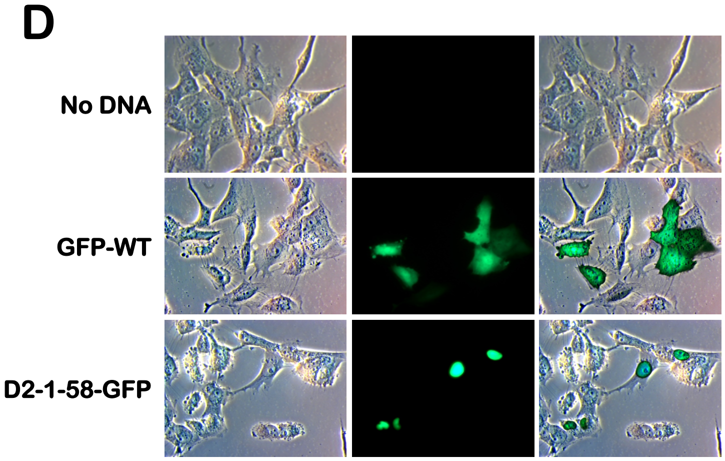

Figure S1D

E

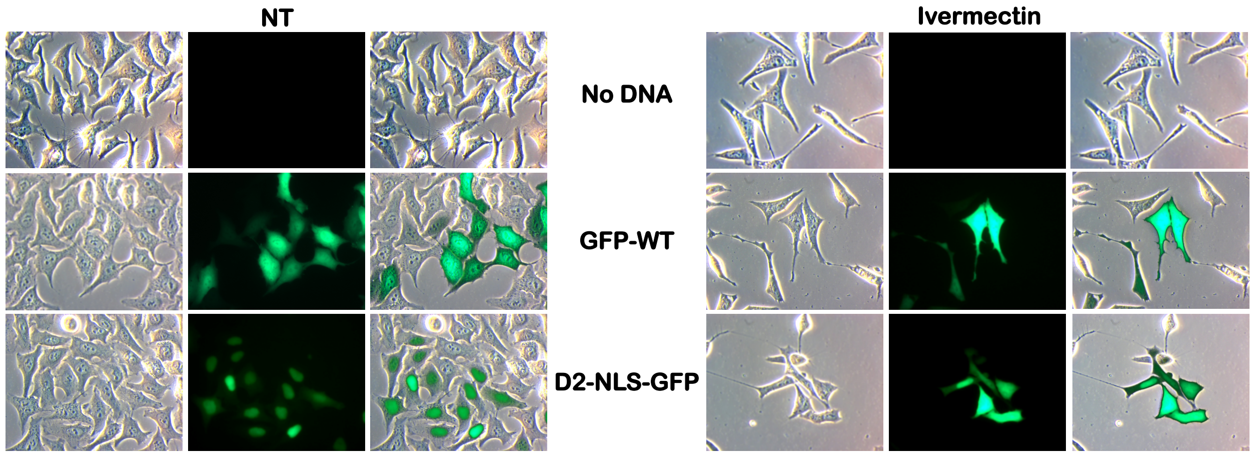

F

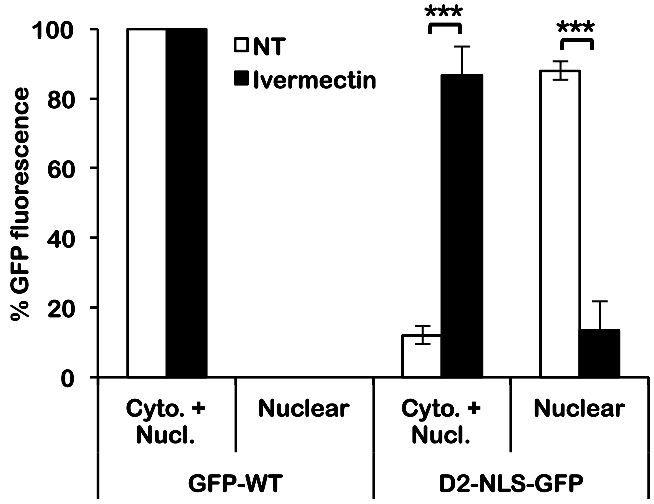

Figures S1E and F

G

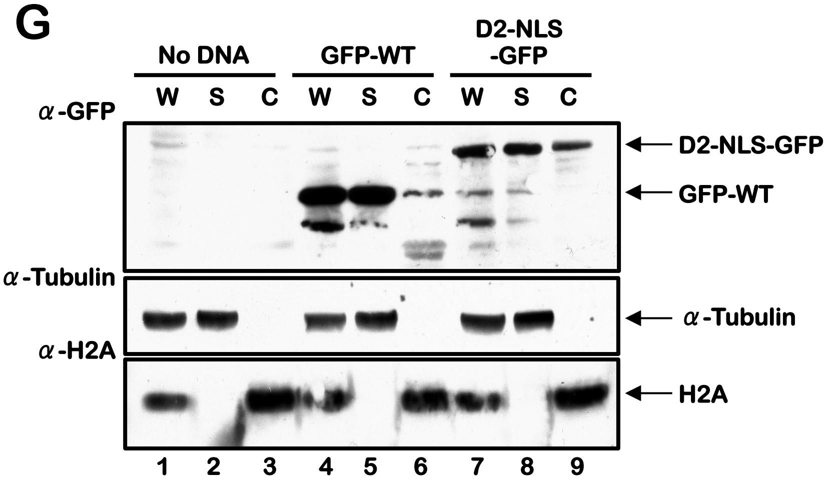

H

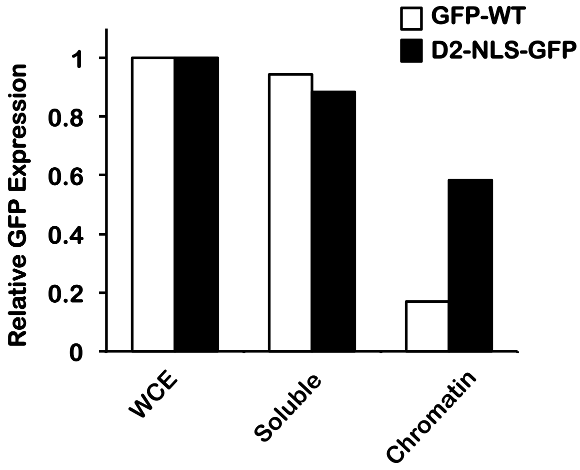

Figures S1G and H
