# Supplementary figures and images for: Coordinate Nuclear Targeting of the FANCD2 and FANCI Proteins via a FANCD2 Nuclear Localization Signal
Source: PLoS One. 2013 Nov 21;8(11):e81387. doi: 10.1371/journal.pone.0081387 (PMC3836817; doi:10.1371/journal.pone.0081387)

**A**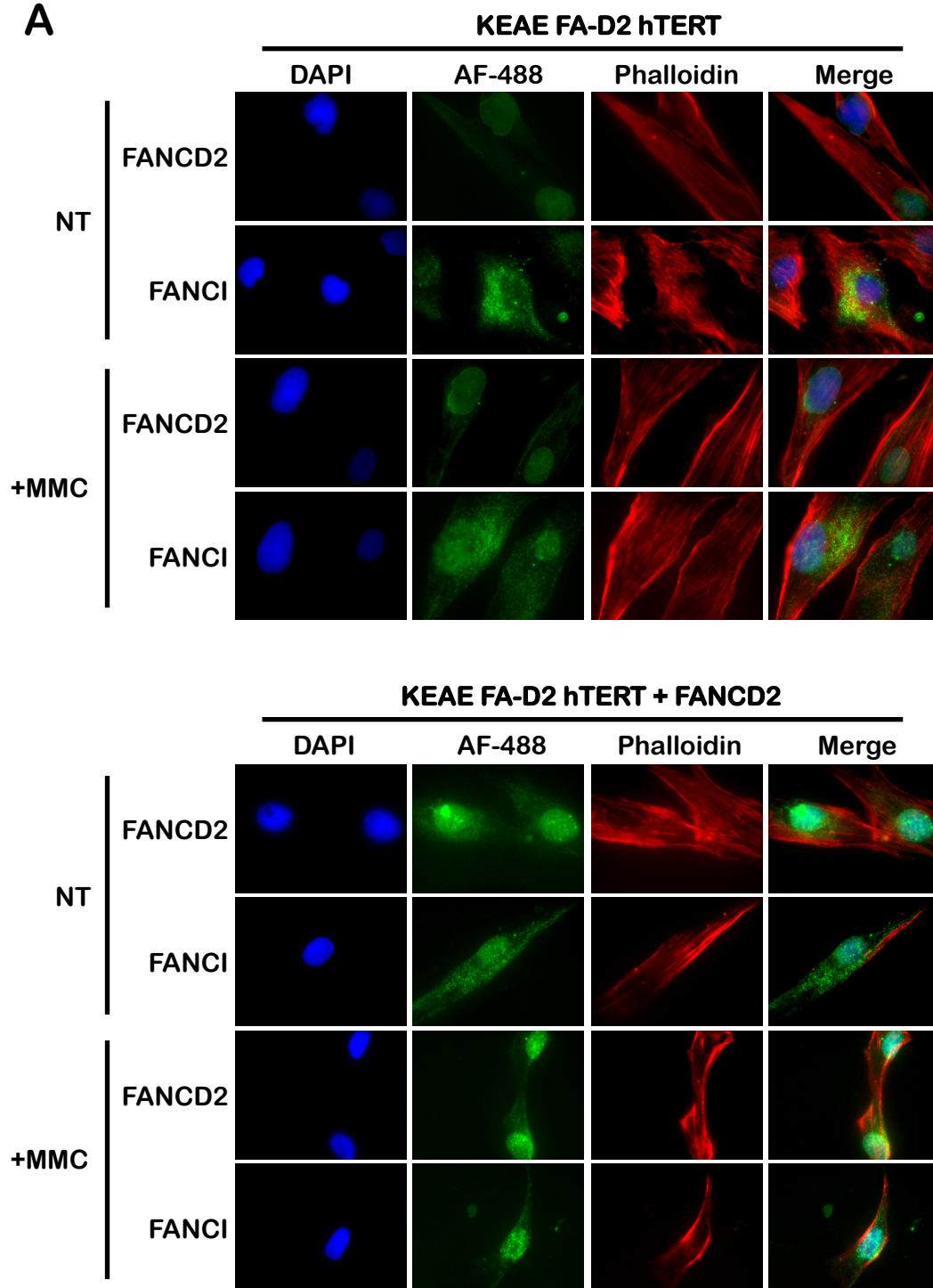**Figure S2A**

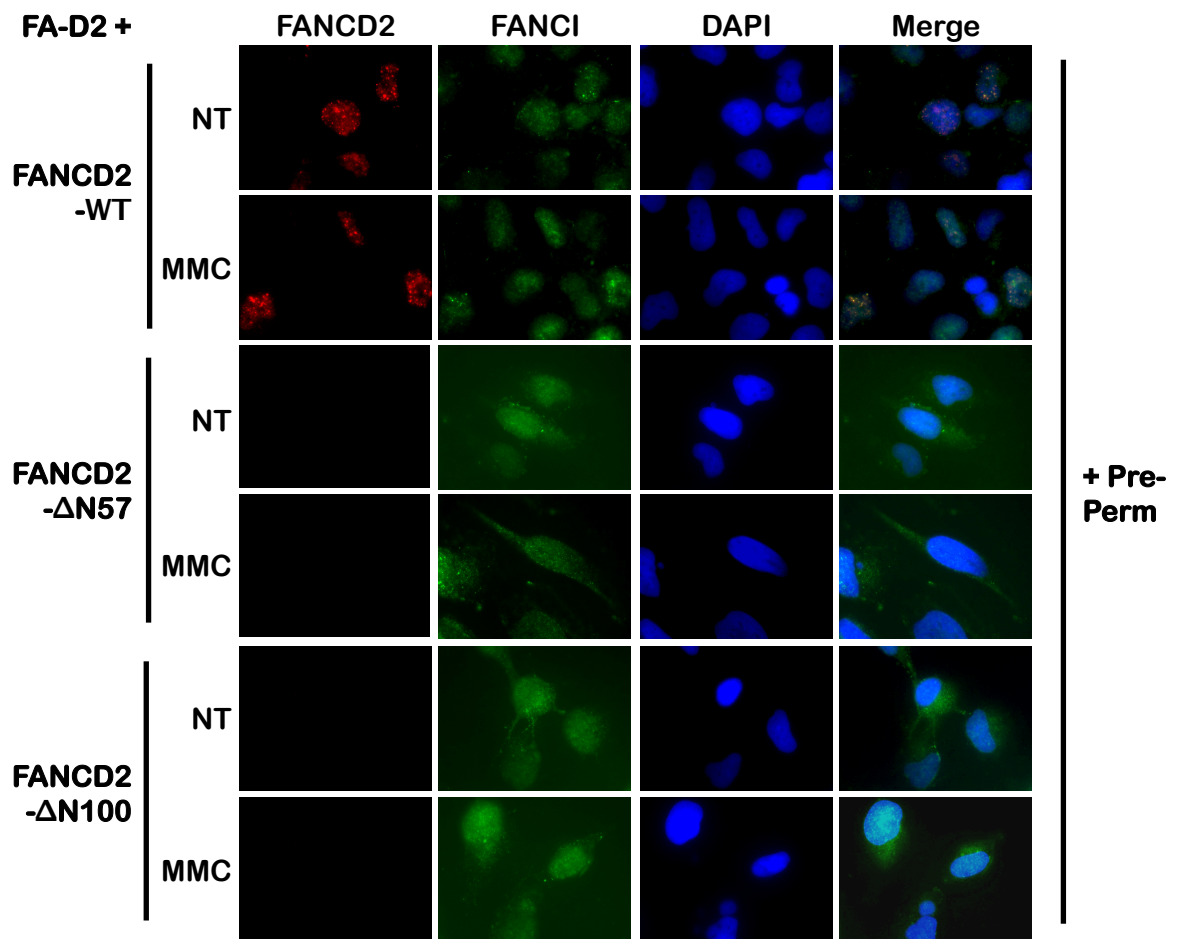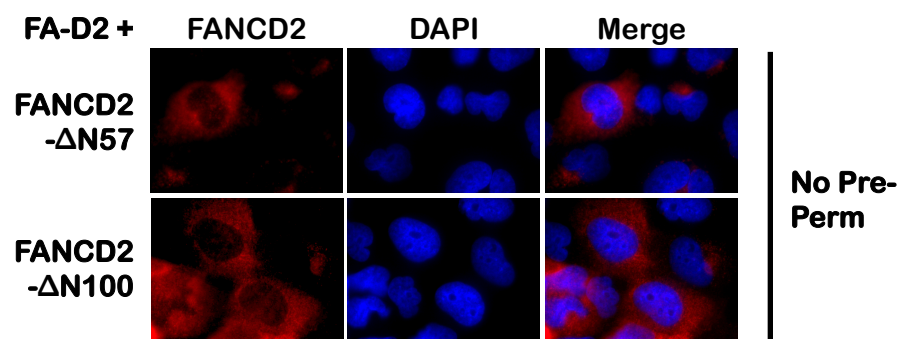

Figure S2B

Supplement: Figure S2 — The FANCD2 NLS is required for the nuclear localization of a subset of FANCI. (A) KEAE FA-D2 hTERT cells or KEAE FA-D2 hTERT cells stably expressing FANCD2-WT were incubated in the absence (NT) or presence of MMC for 24 h, fixed, stained with rabbit polyclonal anti-FANCD2 or anti-FANCI antibody and counterstained with phalloidin and DAPI. AF-488, Alexa Fluor 488. (B) FA-D2 cells stably expressing FANCD2-WT, FANCD2-∆N57, or FANCD2-∆N100 were incubated in the absence (NT) or presence of MMC for 24 h, fixed, stained with mouse monoclonal anti-V5 (red) to detect V5-tagged FANCD2 and rabbit polyclonal anti-FANCI (green) and counterstained with DAPI (blue). IF microscopy was performed with (+ Pre-Perm) and without (No Pre-Perm) a pre-permeabilization step (see Materials and Methods). The pre-permeabilization step leads to complete loss of fluorescent signal for FANCD2-ΔN57 and FANCD2-ΔN100 because of the high solubility of these proteins (see Figure 2B), while this step is necessary for the resolution of FANCI fluorescence signal. (PDF) [file pone.0081387.s002.pdf]

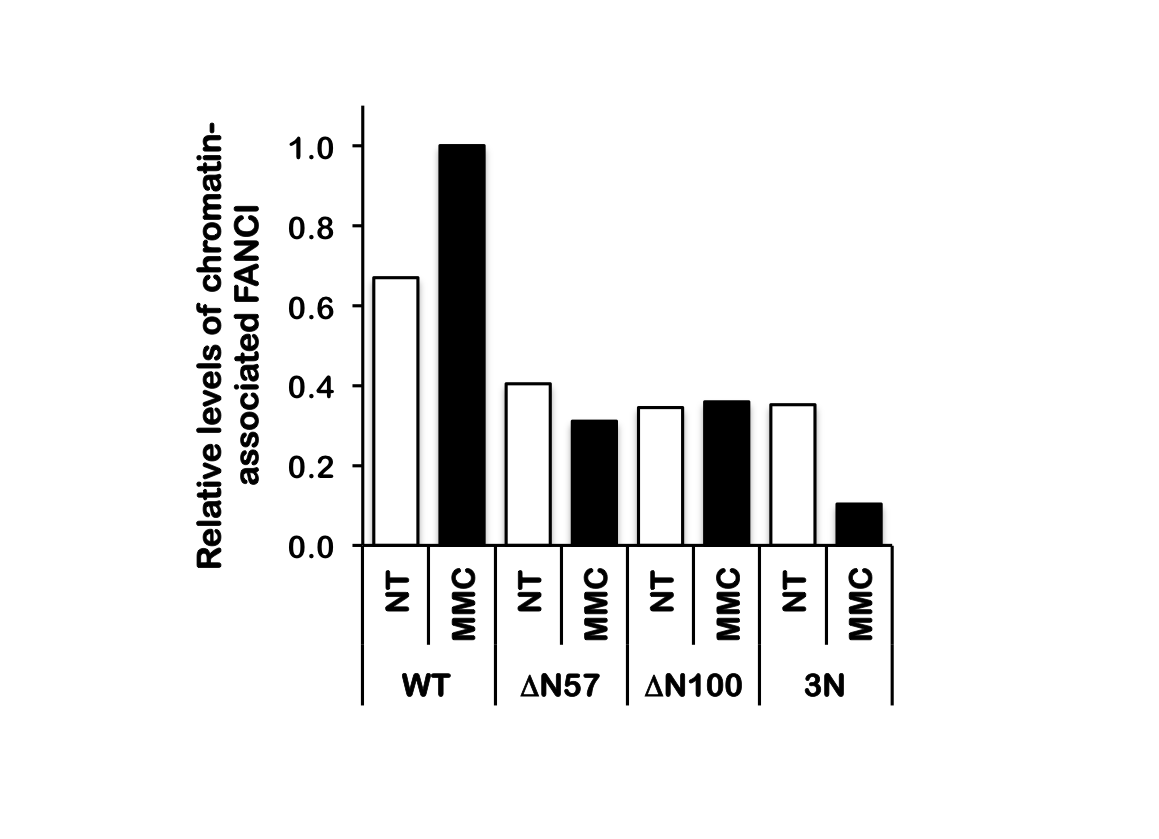

Supplement: Figure S3 — The FANCD2 NLS is required for efficient FANCI chromatin association. FA-D2 cells stably expressing FANCD2-WT (WT), FANCD2-∆N57 (∆N57), FANCD2-∆N100 (∆N100) or FANCD2-3N (3N) were incubated in the absence or presence of MMC for 18 h and cell pellets were fractionated into soluble and chromatin-associated fractions (see Figures 4B and C). The total integrated densities of the chromatin-associated (C) nonubiquitinated and monoubiquitinated FANCI protein bands were quantified using ImageJ image processing and analysis software, and plotted. While the integrated band densities for a single experiment are shown, these experiments were repeated several times with similar findings. NT, not treated; MMC, MMC-treated. (TIF) [file pone.0081387.s003.tif]

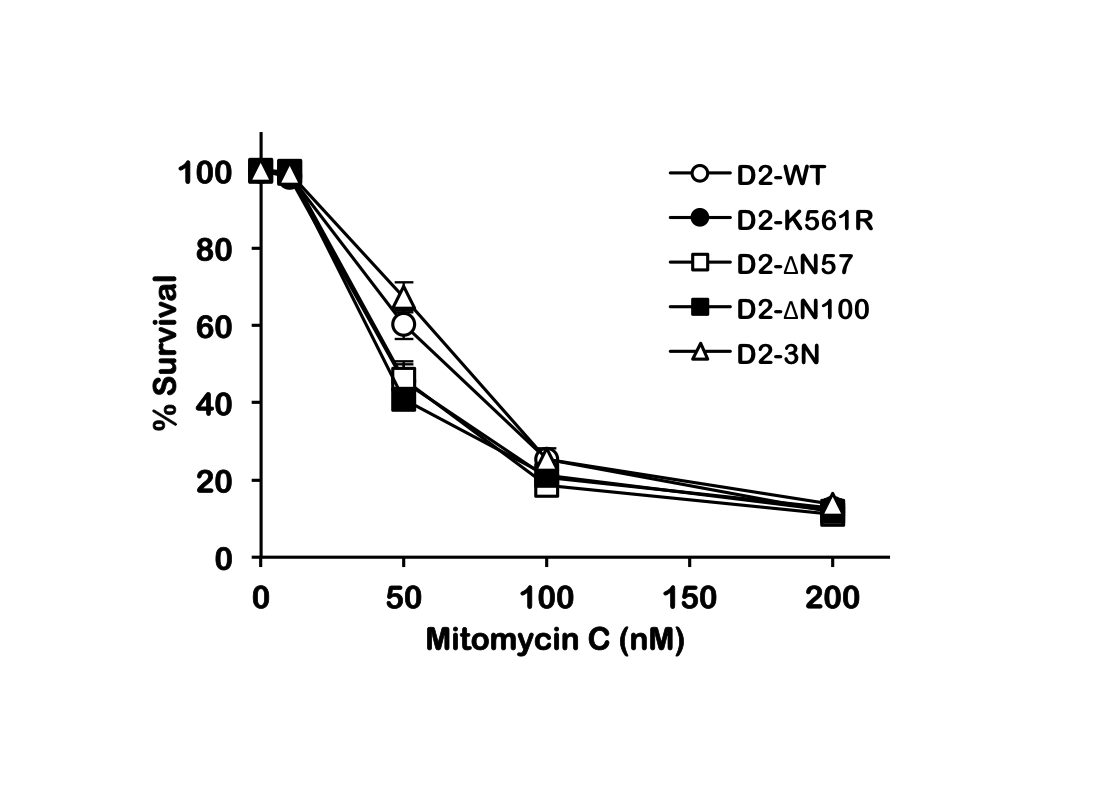

Supplement: Figure S4 — The FANCD2 NLS deletion mutants fail to rescue the MMC sensitivity of FA-D2 cells. FA-D2 cells stably expressing FANCD2-WT, FANCD2-K561R, FANCD2-∆N57, FANCD2-∆N100, or FANCD2-3N were treated with the indicated concentrations of MMC for 7-10 days. Cells were fixed and stained with crystal violet and percent survival calculated and plotted. Each measurement was performed in triplicate and experiments were performed multiple times with similar results. The 20% trimmed means (20%) for all recorded measurements were calculated and plotted. Error bars represent the standard errors of the means. (TIF) [file pone.0081387.s004.tif]

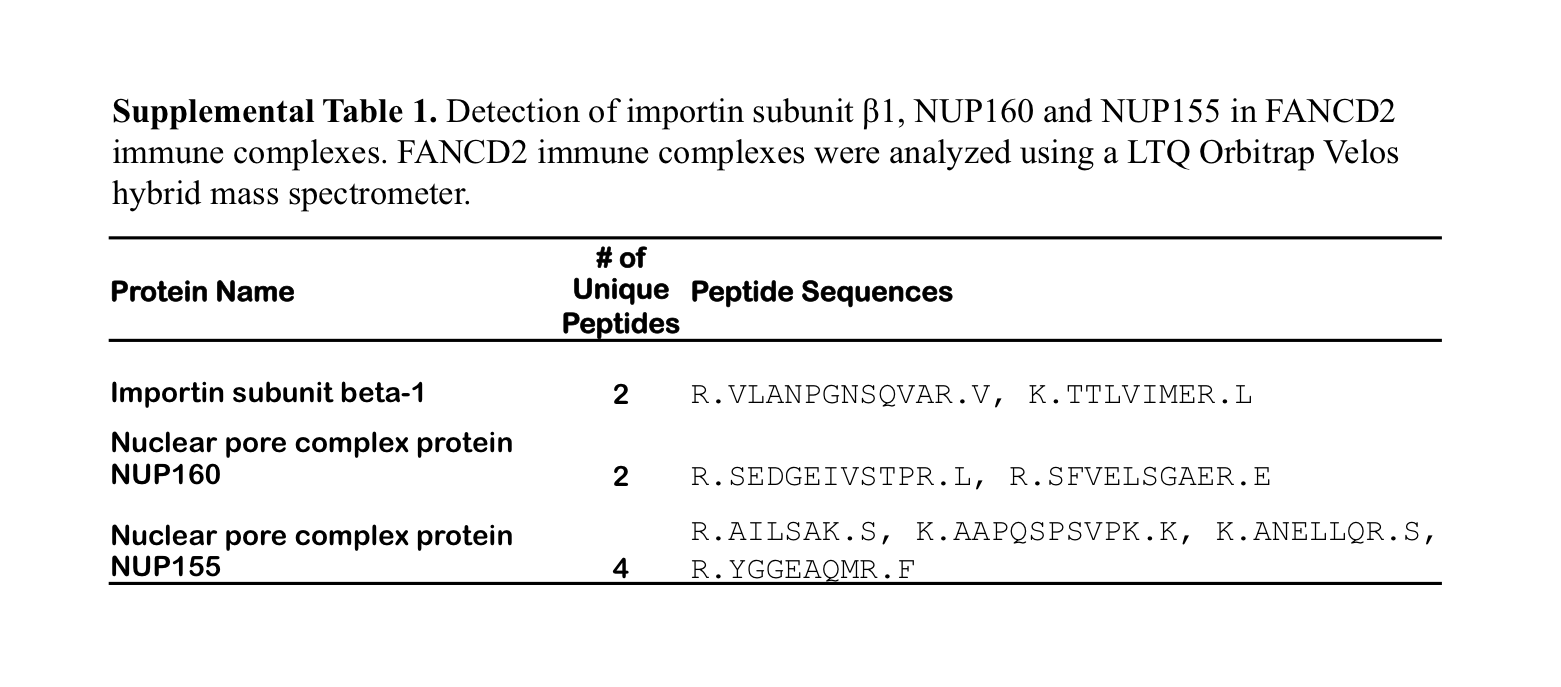

Supplement: Table S1 — Detection of importin subunit β1, NUP160 and NUP155 in FANCD2 immune complexes. FANCD2 immune complexes were analyzed using a LTQ Orbitrap Velos hybrid mass spectrometer. (TIF) [file pone.0081387.s005.tif]
