# Supplementary material for: Coordinate Nuclear Targeting of the FANCD2 and FANCI Proteins via a FANCD2 Nuclear Localization Signal
Source: PLoS One. 2013 Nov 21;8(11):e81387. doi: 10.1371/journal.pone.0081387 (PMC3836817; doi:10.1371/journal.pone.0081387)
Supplement: Methods S1 — (DOCX) [file pone.0081387.s006.docx]

**Supplemental Information Materials and Methods**

**Generation of FANCD2-GFP fusion vectors**

To fuse amino acids 1-27 of FANCD2 to the amino terminus of GFP to generate D2-1-27-GFP, we PCR amplified the coding sequence of amino acids 1-27 using the following forward and reverse primers: FP, 5’-AAAGAGCTCCACCATGGTTTCC-3’; RP, 5’-TATCTGCAGTGGTTGCTTCCTGGT-3’. Similarly, D2-24-55-GFP was generated by PCR amplifying amino acids 24-55 of FANCD2 using the following primers: FP, 5’-ATAGAGCTCCACCATGAGGAAGCAACCACT-3’; RP, 5'-GCGCTGCAGTGATATCTTAAGAAGCTTTACA-3’. D2-1-58-GFP was generated by PCR amplifying amino acids 1-58 of FANCD2 using the following primers: FP, 5’-AAAGAGCTCCACCATGGTTTCC-3’; RP 5’-CCCCTGCAGAATAATTCCTGATATC-3’. All amplified fragments were directionally cloned into the *SacI-PstI* site of pGFP-N1 (Clontech). DNA fragments were amplified using an Eppendorf Mastercycler ep thermal cycler using the following conditions: Thirty cycles of denaturation at 95°C for 30 sec, annealing at 59-65°C for 1

min, and extension at 68°C for 1

min, followed by cooling to 4°C.
